# Epigenetic frontiers: miRNAs, long non-coding RNAs and nanomaterials are pioneering to cancer therapy

**DOI:** 10.1186/s13072-024-00554-6

**Published:** 2024-10-16

**Authors:** Rajkumar Prabhakaran, Rajkumar Thamarai, Sivabalan Sivasamy, Sivanesan Dhandayuthapani, Jyoti Batra, Chinnaperumal Kamaraj, Krishnasamy Karthik, Mohd Asif Shah, Saurav Mallik

**Affiliations:** 1 Central Research Facility, Santosh Deemed to be University, Ghaziabad, UP India; 2 UGC Dr. D.S. Kothari Postdoctoral Fellow, Department of Animal Science, Manonmaniam Sundaranar University, Tirunelveli, Tamil Nadu 627012 India; 3 Interdisciplinary Institute of Indian System of Medicine, Directorate of Research, SRM Institute of Science and Technology, Kattankulathur, Tamil Nadu 603203 India; 4 Department of Mechanical Engineering, Vel Tech Rangarajan Dr. Sagunthala R&D Institute of Science and Technology, Chennai, India; 5 Department of Economics, Kardan University, Parwane Du, 1001 Kabul, Afghanistan; 6 Department of Environmental Health, Harvard T H Chan School of Public Health, Boston, Massachusetts 02115 United States; 7 Department of Pharmacology & Toxicology, University of Arizona, Tucson, AZ 85721 USA; 8 Division of Research and Development, Lovely Professional University, Phagwara, Punjab 144001 India; 9 Centre of Research Impact and Outcome, Chitkara University Institute of Engineering and Technology, Chitkara University, Rajpura, Punjab 140401 India

**Keywords:** Cancer, DNA methylation, Histone modifications, Non-coding RNAs epigenetic therapy, Nanomedicine

## Abstract

Cancer has arisen from both genetic mutations and epigenetic changes, making epigenetics a crucial area of research for innovative cancer prevention and treatment strategies. This dual perspective has propelled epigenetics into the forefront of cancer research. This review highlights the important roles of DNA methylation, histone modifications and non-coding RNAs (ncRNAs), particularly microRNAs (miRNAs) and long non-coding RNAs, which are key regulators of cancer-related gene expression. It explores the potential of epigenetic-based therapies to revolutionize patient outcomes by selectively modulating specific epigenetic markers involved in tumorigenesis. The review examines promising epigenetic biomarkers for early cancer detection and prognosis. It also highlights recent progress in oligonucleotide-based therapies, including antisense oligonucleotides (ASOs) and antimiRs, to precisely modulate epigenetic processes. Furthermore, the concept of epigenetic editing is discussed, providing insight into the future role of precision medicine for cancer patients. The integration of nanomedicine into cancer therapy has been explored and offers innovative approaches to improve therapeutic efficacy. This comprehensive review of recent advances in epigenetic-based cancer therapy seeks to advance the field of precision oncology, ultimately culminating in improved patient outcomes in the fight against cancer.

## Introduction

Cancer, a complex and devastating group of diseases, continues to be a major global health concern. It is characterized by the uncontrolled growth and spread of abnormal cells [1–4] and a leading cause of death worldwide [5–10]. In 2020, there was approximately 10 million cancer-related deaths, continuous  to be second-leading cause of death after cardiovascular diseases [11, 12]. The number of new cancer cases diagnosed each year worldwide was estimated to be over 19 million [13]. This number is expected to continue to rise due to factors such as elderly peoples and lifestyle changes [11, 13]. In recent years, a growing focus on the significance of epigenetics in cancer initiation, progression, therapeutic approaches and significant strides have been made in comprehending the genetic changes associated with different types of cancer [7, 13, 14]. Epigenetic modifications like DNA methylation, histone alterations, and non-coding RNAs control genetic material expression and chromatin structure. Dysregulation of these mechanisms links to aberrant gene expression, impacting key pathways in oncogenesis [15–26]. Specific epigenetic changes, like DNA hypermethylation in tumor suppressor genes or global hypomethylation, underlie various cancers, disrupting signaling pathways and DNA repair, and influencing immune response genes [27, 28]. The identification of epigenetic biomarkers has gained significant momentum in recent years [29–32]. Researchers have focused on identifying DNA methylation patterns or histone modifications that are specific to certain cancers. These epigenetic signatures have the potential to revolutionize cancer diagnostics, enabling early detection and personalized treatment strategies [29]. The development of epigenetic therapies has emerged as a promising avenue in cancer treatment. Epigenetic drugs, such as DNA methyltransferase inhibitors and histone deacetylase inhibitors, have shown therapeutic efficacy in various preclinical and clinical studies. These therapies are used to reverse aberrant epigenetic modifications, restoring normal gene expression patterns and halting cancer progression [30–35].

Recently, the effectiveness of nanoparticle-based epigenetic medication delivery is its capacity to capitalize on the tumor vasculature’s increased permeability and retention (EPR) effect [40–43]. These nanoparticles can also be modified on the surface and fitted with ligands to enable active targeting, which increases their specificity for cancer cells. This approach holds great promise for improving cancer patient outcomes [30]. The role of non-coding RNAs, including microRNAs and long non-coding RNAs, in cancer epigenetics has gained significant attention. Understanding the intricate interplay between non-coding RNAs and epigenetic modifications represents a cutting-edge area of cancer research [2]. This review aims to address two distinct yet interconnected areas: the impact of epigenetic modifications on cancer and the application of nanomaterials in cancer therapy. Specifically, this review explores, influence of DNA methylation, histone modifications, and non-coding RNAs to cancer progression and targeting these epigenetic alterations to offer new therapeutic strategies. Additionally, the current review examines the role of nanomedicine, particularly nanoparticle-based delivery systems, in enhancing the effectiveness of epigenetic therapies through mechanisms such as the enhanced permeability and retention (EPR) effect and active targeting.

## Understanding cancer epidemiology

Cancer continues to be a significant global health concern [12]. The leading causes of cancer-related deaths remained consistent, with lung, liver, stomach, breast, and colon malignancies topping the list [36].

The male and female cancer mortality rate in the world is shown in Fig. S1. Mongolia had the highest cancer-related mortality rate among men, with 224.3 deaths per 100,000 individuals (https://www.wcrf.org/cancer-trends/global-cancer-data-by-country/). Epigenetic alterations significantly influence the incidence of several prominent cancers worldwide. Among these, lung cancer stands as the most prevalent, with an estimated annual occurrence of approximately 2.2 million new cases. Following closely, breast cancer registers around 2.3 million new cases annually, while colon cancer contributes roughly 1.9 million new diagnoses each year. Stomach cancer demonstrates an annual incidence of about 1.1 million cases, and liver cancer accounts for approximately 900,000 new cases per year. The impact of epigenetic changes in the development and prevalence of these malignancies is substantially growing.

Numerous factors contribute to cancer prevalence, including high rates of tobacco consumption, occupational exposure to specific chemicals, infectious agents like HPV and hepatitis B/C, exposure to environmental toxins, and limited access to healthcare facilities, particularly in rural areas (Table 1). Bladder cancer emerged prominently, study led by Ibrahim Jubber, reporting 573,000 cases worldwide, resulting in 213,000 fatalities. Möller et al.’s study analyzed 167,919 cases, with adenocarcinoma prevailing at 86.4% [37]. Notable disparities were observed in age-standardized incidence rates across age brackets. Incidence dropped among older individuals but remained relatively steady in the younger group. Neuroendocrine tumors, T1 tumors, and G2 tumors exhibited an alarming increase in both age groups.

**Table 1:** Primary risk factors linked to each type of cancer

Cancer type	Risk factors
Lung cancer	Smoking, exposure to asbestos, radon, air pollution
Breast cancer	Genetic mutations (BRCA genes), hormonal factors
Colon cancer	Diet (low fiber, high fat), genetic predisposition
Stomach cancer	Helicobacter pylori infection, smoking, diet
Liver cancer	Chronic viral hepatitis (HBV, HCV), alcohol consumption

Furthermore, younger patients exhibited higher relative survival rates, particularly in neuroendocrine tumors. Mederos’s study highlighted evolving gender disparities in lung cancer [38]. Meanwhile, Abood et al. research in Basra found leukemia to be the leading cause of child mortality, while urinary bladder, lung, and bronchus cancers prevailed in adult males [39]. The study emphasized the significance of understanding demographics for effective cancer prevention and treatment. It is imperative to disseminate this information to empower healthcare professionals, researchers, and the public in the ongoing battle against cancer, exploring innovative approaches like epigenetics and nano-medicine for more effective prevention and treatment strategies.

### Global incidence and prevalence of different cancers

Cancer remains a formidable global health challenge, marked by varying incidence and prevalence rates across different regions. The proportion of new cancer cases in less developed countries is expected to rise from approximately 56% in 2008 to over 60% by 2030, driven by increasing cancer rates, improvements in life expectancy, and population growth [151]. An extensive overview reveals lung cancer as the most frequently diagnosed worldwide, accounting for about 2.2 million new cases annually [152]. High incidence rates are seen in countries with significant tobacco use and air pollution, such as the United States, China, and Australia [152]. Lung cancer also carries the highest mortality rate, notably affecting Eastern Europe, Central Asia, and North America [153]. Breast cancer ranks as the most prevalent cancer among women globally, with approximately 2.3 million new cases annually [154]. Higher incidence rates are evident in North America, Western Europe, and Australia, reflecting advanced detection capabilities and actual disease prevalence. Mortality rates are particularly pronounced in regions with limited access to early detection and treatment, including parts of Africa and South Asia [154]. Colon cancer, with an annual incidence of around 1.9 million cases globally, shows peak rates in North America, Europe, and Australia, influenced by dietary and lifestyle factors [155, 156]. Stomach cancer, responsible for about 1.1 million new cases per year, is most prevalent in East Asia, especially in Japan and South Korea, due to dietary practices and *Helicobacter pylori* infection [157]. Liver cancer affects approximately 900,000 individuals annually, with the highest incidence rates in East Asia and sub-Saharan Africa, linked to chronic viral hepatitis and aflatoxin exposure [158–160]. Bladder cancer, with approximately 573,000 new cases each year, demonstrates high incidence in North America and Europe, associated with smoking and occupational exposures. Leukemia, a significant global concern, disproportionately impacts regions with varying healthcare infrastructure and access to treatment. Overall, cancer incidence and mortality rates exhibit substantial regional disparities influenced by lifestyle choices, healthcare accessibility, and environmental factors.

## Epigenetic machinery: orchestrating gene regulation and cellular identity

**Fig. 1:**
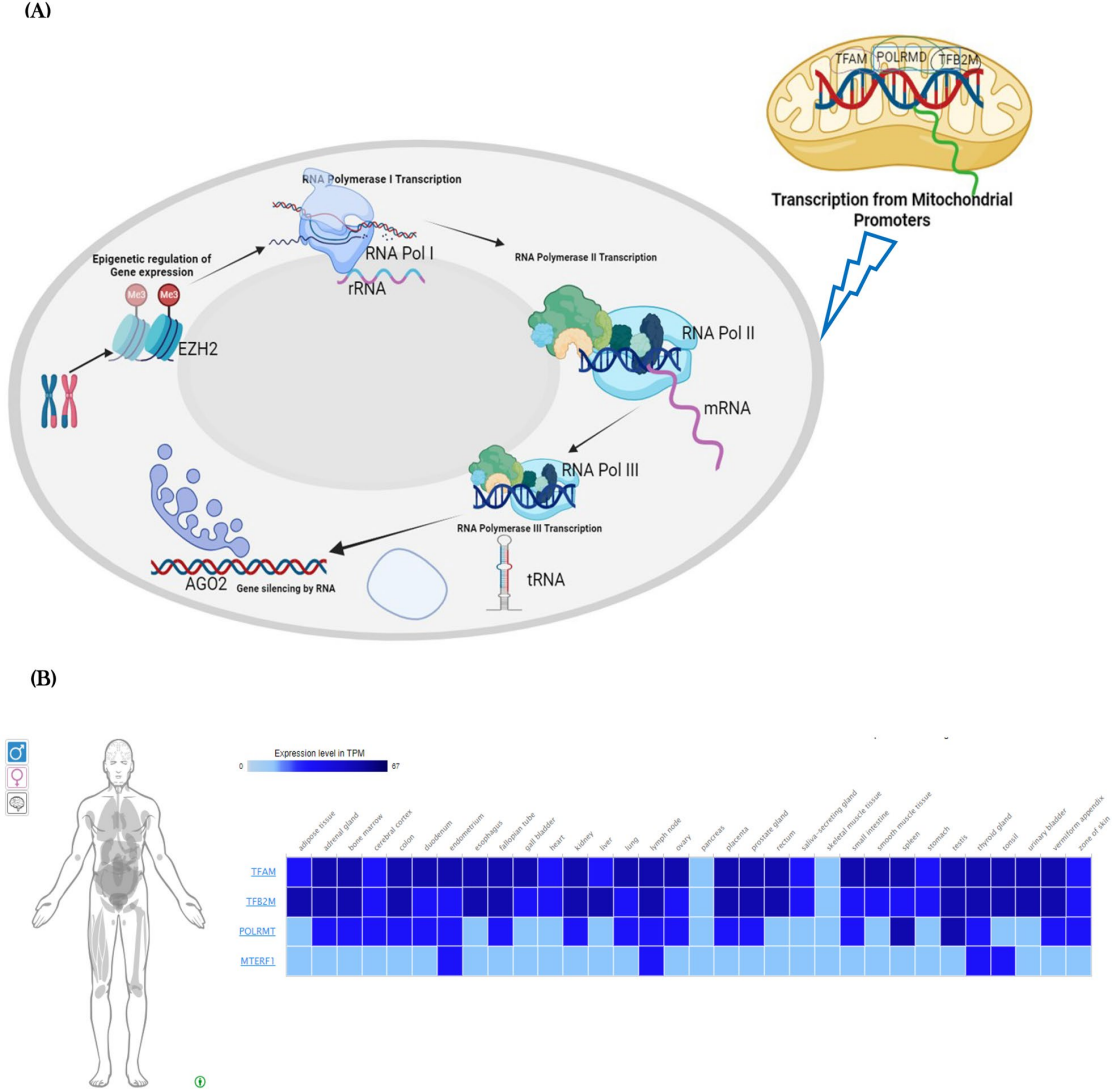
**A** Schematic diagram illustrating the pathway and expression of miRNA21. This figure shows the interaction of miRNA21 within nucleosomes and its role in the regulation of transcription from mitochondrial promoters. Key components include miRNA21, nucleosome complexes, and mitochondrial transcription factors. The pathways and interactions are detailed to illustrate how miRNA21 influences gene expression at the chromatin level. **B** Schematic diagram showing the expression of miRNA21 across different tissues. The figure integrates RNA-seq data from 122 human individuals representing 32 different tissues, highlighting the differential expression of miRNA21. The diagram includes details on how miRNA21 expression is mapped across various tissue types, with annotations indicating specific tissues and expression levels. For further details, refer to the RNA-seq dataset available at (https://reactome.org/PathwayBrowser/#/R-HSA-74160&SEL=R-HSA-75944&DTAB=EX) [49]

The epigenetic machinery is a complex and highly regulated system and plays a pivotal role in controlling gene expression and cellular identity. It includes three unified components: DNA methylation, histone post-translational modifications, and non-coding RNAs (ncRNAs) [40–43]. These components work together to orchestrate gene regulation and epigenetic modifications, ultimately shaping an organism's development, cellular differentiation, and response to environmental cues (Fig. 1a, b).

Within cellular differentiation, pivotal players like PRC1 and PRC2, belonging to polycomb complexes, orchestrate specific gene expression patterns. PRC1, recognizing and binding to the H3K27me3 mark, further fortifies the repressive state by introducing ubiquitin molecules to histone H2A. PRC2 assumes responsibility for adding methyl groups to histones and DNA, instigating signals that lead to repression, leading to the formation of trimethylated lysine-27 on histone H3 (H3K27me3) as well as 5-methylcytosine within DNA. This marks the groundwork for gene repression. Eukaryotic cells rely on three nuclear RNA polymerases, each with distinct functions. RNA polymerase thorough module dives into the realm of regulatory non-coding RNAs, explicating their biogenesis and functions. It spans the mechanisms involved in generating small interfering RNAs (siRNAs), microRNAs (miRNAs), PIWI-interacting small RNAs (piRNAs), and tRNA-derived small RNAs (tsRNAs), elucidating their diverse roles in cellular regulation.

Mitochondrial genetics present an intriguing landscape, with approximately 80 proteins comprising the human mitochondrial respiratory chain. Thirteen of these proteins derive from the circular mitochondrial genome (mtDNA), which exists in cells in varying copies, ranging from 1000 to 10,000. Alongside encoding proteins, mtDNA contains two rRNAs as well as twenty-two tRNAs, deficient introns within its double-stranded structure. Important components governing transcription and replication are located in the non-coding region of mtDNA, highlighting the non-coding region’s critical role in mitochondrial upkeep and function. Recent studies have demonstrated the important role that epigenetic changes, such as modifications to histones, methylation of DNA, and non-coding RNAs, play in initiating, advancing, and metastasizing various types of cancer [1, 26, 31, 33].

## Methylation of DNA

DNA methylation is the process of adding a methyl group (-CH3) to cytosine residues in DNA molecules, primarily occurring at CpG (cytosine-phosphate-guanine) dinucleotides [44]. DNA methylation, a pivotal modification, is orchestrated by DNA methyltransferase (DNMT) enzymes [32, 44, 45]. While the de novo methyltransferases *DNMT3A* and *3B* target hemimethylated or unmethylated CpG sites, initiating novel methylation patterns, *DNMT1* plays the critical role of maintaining pre-existing DNA methylation [32, 46].

This epigenetic phenomenon exerts profound influence over a plethora of vital biological processes in the mammalian genome. They include gene expression and post-transcriptional processing control, coordination of post-translational modifications, chromatin structure modification, control over genomic imprinting, X chromosome inactivation, and suppression of repetitive DNA sequences. [35, 44, 45]. DNA methylation is responsible for maintaining genomic stability, silencing repetitive elements, and regulating tissue-specific gene expression patterns [32, 35, 44–46]. Sun et al. examined the impact of a catalytic activity-defective mutant and wild-type (WT) UTX on the gene expression profiles of 786-O and HCT116 cells. The bulk of target genes’ expression is significantly regulated by a mix of catalytic activity dependent and independent mechanisms, as the study confirmed. These results highlight the possibility of creating and applying pharmaceuticals that specifically target alterations in H3K27 or H3K4 for therapeutic purposes [47]. Epigenetic modifications exert their influence on genes critical for tumor suppression, DNA repair, and the regulation of the cell cycle [24, 25, 34, 48].

## Epigenetic modifications in cancer: impact and implications

Cancer epimutations involve the hypermethylation of CpG islands within the promoter regions of tumor suppressor genes [34]. This hypermethylation effectively silences p53, RB1, BRCA1, MLH1 and VHL pivotal genes, unleashing unbridled cell growth. Furthermore, the phenomenon of global hypomethylation, prevalent in many cancer types, can give rise to genomic instability and the activation of oncogenes. Additionally, these changes play a pivotal role in the emergence of drug resistance in cancer cells [24, 34, 48]. For instance, alterations in epigenetic marks can lead to modified expression of drug transporters and DNA repair enzymes, rendering the cancer cells less susceptible to treatment.

Recent studies have revealed the involvement of hydroxymethylation in cancer development [32, 35, 50]. This process actively participates in DNA demethylation by oxidizing 5-methylcytosine (5-mC) to 5-hydroxymethylcytosine (5-hmC), catalyzed by a methylcytosine oxygenase and involving members of the TET protein family [26]. Reduced expression of TET genes in human cancers leads to diminished levels of hydroxymethylation [31, 35, 51]. Additionally, N6-methyladenine (N6-mA) alterations, functioning as a repressive epigenetic marker beyond 5-mC and 5-hmC methylation states, suppress long interspersed nuclear element (LINE) transposons. Recent studies have observed elevated N6-mA levels in glioblastoma [33, 52].

## Histone post-translational modification: bridging genetics and epigenetics

Histone post-translational modifications encompass chemical alterations (e.g., acetylation, methylation, and phosphorylation) to histone tails [45, 52, 53]. These modifications dynamically influence chromatin structure, affecting its accessibility to transcription factors and RNA polymerase, thereby regulating gene expression [33, 45, 46, 50]. This ‘histone code’ determines gene activity or silencing, responding to cellular signals [32]. Histone proteins, forming the core of nucleosomes, consist of a structured C-terminal domain and an unstructured N-terminal tail [32, 53]. These tails undergo diverse post-translational covalent alterations, including methylation, acetylation, ubiquitylation, sumoylation, and phosphorylation at specific residues. Zhao and Shilatifard have outlined additional alterations, such as lactylation, deimination, ubiquitylation, ADP-ribosylation, deamination, formylation, O-GlcNAcylation, propionylation, butyrylation, crotonylation, and proline isomerization [53].

These modifications are central to regulating essential cellular processes like transcription, replication, and repair. Together, they constitute the 'histone code,’ preserving cellular memory and dictating chromatin structure and function. Their impact extends to chromatin accessibility and their ability to recruit or sequester non-histone effector proteins, interpreting encoded information patterns.

## Non-coding RNA: microRNAs (miRNAs) and long non-coding RNAs (lncRNAs)

Transcribed RNAs, predominantly comprising non-coding sequences, include a prominent category known as long non-coding RNAs (lncRNAs) [1, 54]. These transcripts, exceeding 200 nucleotides in length, lack protein-coding potential and are subject to various epigenetic modifications, such as N6-methyladenosine, N1-methyladenosine, 5-methylcytosine, 7-methylguanosine, and 2′-O-methylation [55]. Enzymes categorized as ‘writers,’ ‘readers,’ and ‘erasers’ orchestrate these RNA modifications within the epigenetic framework [1, 54, 56]. Among these modifications, m6A and m5C have garnered significant scientific interest, leading to structural and functional alterations in lncRNAs [57]. An overview of different types of small RNA molecules and their roles in gene regulation at various levels were shown in Fig. S2 (a, b).

**Fig. 2:**
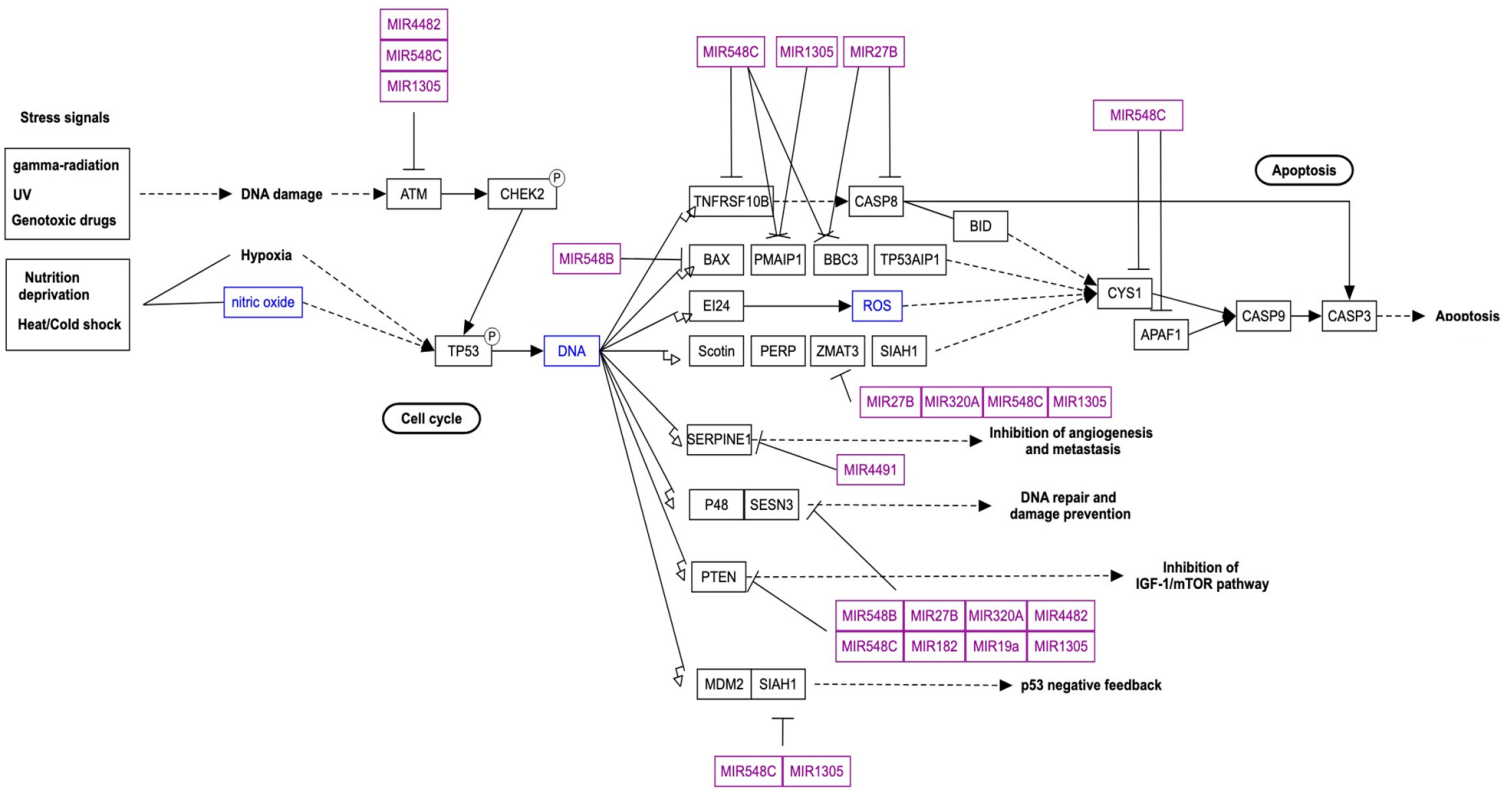
Diagram illustrating the regulation of the p53 pathway by miRNAs in prostate cancer (WP3982). The figure depicts how elevated levels of specific miRNAs affect the p53 signaling pathway in prostate cancer cells. Key elements shown include the targeted genes within the p53 pathway, the interactions between miRNAs and these genes, and the overall impact on pathway activity. For a detailed view of the pathway, refer to (https://www.wikipathways.org/pathways/WP3982.html)

Non-coding RNAs encompass a diverse range of RNA molecules that play pivotal roles in gene regulation without encoding proteins. The major classes within this domain include miRNAs and lncRNAs [58]. The miRNAs, a type of small non-coding RNA, are crucial for posttranscriptional gene expression regulation, impacting various biological functions like cell division, proliferation, and programmed cell death [59]. The miRNAs have been associated with numerous diseases, and ongoing clinical trials utilizing miRNA-based approaches have shown promise in treating conditions such as cancer and viral infections [52, 53, 58, 59]. These brief RNA molecules interact with messenger RNAs (mRNAs), leading to mRNA degradation or translational repression. Figure 2 elucidates the miRNA-mediated regulation of the p53 pathway in prostate cancer (WP3982), highlighting their role as post-transcriptional gene expression regulators.

**Fig. 3:**
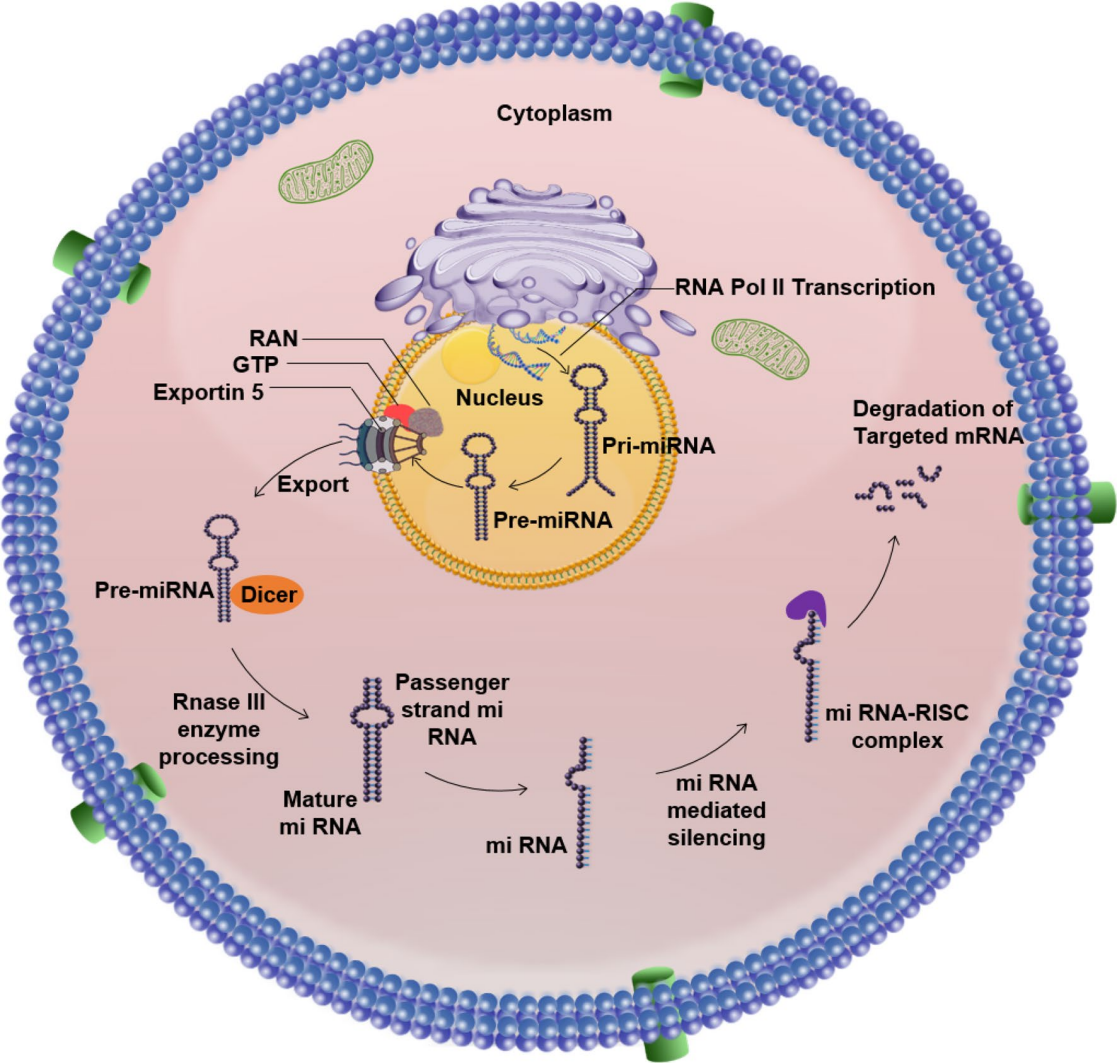
The schematic representation reveals the biogenesis of microRNAs (miRNAs). The biogenesis of microRNAs (miRNAs) unfolds through five key stages: Transcription: miRNA precursors originate from autonomously transcribed genes, co-transcripts with other genes, or introns of host genes. RNA polymerase II transcribes most miRNAs, though some come from RNA polymerase III co-transcripts with adjacent repetitive elements. The initial transcript, known as primary microRNA (pri-miRNA), includes an imperfectly double-stranded region within a hairpin loop, with longer sequences extending from both the 5′ and 3′ ends. Cleavage by DROSHA: The DROSHA nuclease, in association with the RNA-binding protein DGCR8 (forming the Microprocessor complex), endoribonucleolytically cleaves the 5′ and 3′ ends of the pri-miRNA. This cleavage produces a short hairpin structure, about 60 to 70 nucleotides long, called pre-microRNA (pre-miRNA). Nuclear Export by Exportin-5: The pre-miRNA associates with Exportin-5, Ran, and GTP to be transported through the nuclear pore into the cytoplasm. Cleavage by DICER1: In the cytoplasm, the pre-miRNA is processed by the RISC loading complex, which includes DICER1, an Argonaute protein, and either TARBP2 or PRKRA. DICER1 cleaves the pre-miRNA, resulting in a double-stranded miRNA approximately 21 to 23 nucleotides in length, with protruding single-stranded 3′ ends of 2–3 nucleotides. Incorporation into RNA-Induced Silencing Complex (RISC) and Strand Selection: The double-stranded miRNA is incorporated into an Argonaute protein within the RISC loading complex. The passenger strand is removed and degraded, while the guide strand is retained, directing the Argonaute complex (RISC) to target mRNAs

The Fig. 3 clearly reveals the biogenesis of microRNAs (miRNAs). The biogenesis of microRNAs (miRNAs) can be succinctly outlined in five stages. List of well-studied miRNAs associated with various types of cancer was shown Table 2.

**Table 2:** List of well-studied miRNAs associated with various types of cancer.** (**http://mirwalk.umm.uni-heidelberg.de/human/mirna/MIMAT0000437/)

mRNAs	Function	Related disease	Sequence (with Refseqid)	Effects	Conclusions	Reference
miR-21	Oncogenic miRNA	Elevated levels in various cancers, including breast, colorectal, lung, and pancreatic cancer	UAGCUUAUCAGACUGAUGUUGA (NM_015000)	Promotes tumor growth and survival by targeting tumor suppressor genes	Potential therapeutic target due to its role in multiple cancer types	[68–71]
miR-34a	Tumor suppressor miRNA	Downregulated in multiple cancers, including lung, breast, and prostate cancer	UGGCAGUGUCUUAGCUGGUUGU (NM_001256426)	Inhibits tumor growth and promotes apoptosis; loss contributes to cancer progression	Restoration of miR-34a levels could suppress tumor growth	[10, 72]
miR-155	Oncogenic miRNA	Overexpressed in lymphoma, leukemia, breast, lung, and pancreatic cancer	UUAAUGCUAAUCGUGAUAGGGGUU(NM_001271900)	Enhances cancer cell proliferation and survival by targeting tumor suppressors	A promising target for cancer therapy, especially in hematological malignancies	[73]
miR-200 family (miR-200a, miR-200b, miR-200c, miR-141, miR-429)	Tumor suppressor miRNAs	Involved in epithelial-mesenchymal transition (EMT) and metastasis in various cancers	CAUCUUACUGGGCAGCAUUGGA (NM_004136)	Regulates EMT and metastasis; downregulation facilitates cancer progression	Reinstating miR-200 expression might inhibit metastasis	[74]
miR-17-92 cluster	Oncogenic miRNA cluster	Overexpression in several cancers, including lymphoma, lung, and breast cancer	CAAAGUGCUUACAGUGCAGGUAG (NM_001366280)	Promotes cell proliferation and survival; dysregulation contributes to tumorigenesis	Potential target for interventions aimed at multiple cancer types	[75]
let-7 family (let-7a, let-7b, let-7c)	Tumor suppressor miRNAs	Downregulated in various cancers, including lung, ovarian, and colorectal cancer	UGAGGUAGUAGGUUGUAUAGUU (NM_001348204) UGAGGUAGUAGGUUGUGUGGUU (NM_015279) UGAGGUAGUAGGUUGUAUGGUU (NM_001330410)	Inhibits tumor growth; loss of let-7 contributes to increased tumorigenesis	Restoration of let-7 levels could have therapeutic benefits	[76]
miR-221/222	Oncogenic miRNAs	Elevated levels in glioblastoma, breast, and hepatocellular carcinoma	ACCUGGCAUACAAUGUAGAUUU(NM_001268284) CUCAGUAGCCAGUGUAGAUCCU(NM_001256426)	Promotes tumor growth and metastasis by targeting multiple tumor suppressor genes	Targeting miR-221/222 may help in treating various cancers	[77]
miR-10b	Oncogenic miRNA	Associated with invasion and metastasis in breast cancer	UACCCUGUAGAUCCGAAUUUGUG(NM_001256426)	Enhances invasion and metastasis; plays a role in cancer progression	A potential therapeutic target for preventing cancer metastasis	[78]
miR-143/145	Tumor suppressor miRNAs	Downregulated in colorectal and other cancers	GUCCAGUUUUCCCAGGAAUCCCU(NM_032359)	Inhibits cancer cell proliferation and promotes apoptosis	Restoring miR-143/145 levels could suppress tumor growth	[79]
miR-210	Oncogenic miRNA	Overexpressed in various cancers, including lung, breast, and renal cell carcinoma	AGCCCCUGCCCACCGCACACUG(NM_013321)	Enhances tumor survival and resistance to hypoxia; contributes to cancer progression	Potential therapeutic target due to its role in multiple cancer types	[80]
miR-126	Tumor suppressor miRNA	Downregulated in several cancers, including lung, breast, and pancreatic cancer	CAUUAUUACUUUUGGUACGCG(NM_001256549)	Inhibits tumor angiogenesis and proliferation; its downregulation promotes cancer	Reinstating miR-126 may suppress tumor growth and angiogenesis	[81]
miR-221/222	Oncogenic miRNAs	Elevated levels in glioblastoma, breast, and hepatocellular carcinoma	ACCUGGCAUACAAUGUAGAUUU(NM_001268284) CUCAGUAGCCAGUGUAGAUCCU(NM_001256426)	Promotes tumor growth and metastasis by targeting multiple tumor suppressor genes		[82]
miR-31	Oncogenic miRNA	Associated with metastasis in colorectal and breast cancer	AGGCAAGAUGCUGGCAUAGCU(NM_001256426)	Promotes metastasis by influencing cancer cell migration and invasion	Potential target for therapies aimed at reducing cancer metastasis	[83]
miR-29 family (miR-29a, miR-29b, miR-29c)	Tumor suppressor miRNAs	Downregulated in various cancers, including Intra-Hepatic Cholangiocarcinoma, leukemia, lung, and pancreatic cancer	ACUGAUUUCUUUUGGUGUUCAG (NM_001256793)	Regulates cell proliferation, apoptosis, and differentiation	Restoration of miR-29 levels could be beneficial in treating various cancers	[84]

*miRNA* MicroRNA, *Refseqid* Reference Sequence Identifier

### Integrating chromatin accessibility into the epigenetic regulation framework: DNA methylation, histone modifications, and noncoding RNAs

Epigenetic regulation involves complex interactions between DNA methylation, histone modifications, and noncoding RNAs, all of which affect chromatin structure and function [145, 146]. Chromatin accessibility, which determines how readily DNA can be accessed by transcriptional machinery, is a critical factor in this regulatory framework.

### DNA methylation and chromatin accessibility

*Role in Chromatin Accessibility* DNA methylation predominantly occurs at cytosine residues within CpG dinucleotides. This modification is closely associated with transcriptional repression. Methylation of promoter regions can inhibit the binding of transcription factors and other regulatory proteins, leading to reduced chromatin accessibility. Consequently, the DNA becomes less accessible for transcriptional machinery, resulting in gene silencing [147].

*Interaction with Chromatin Structure* Highly methylated regions are often found in heterochromatin, which is a more condensed and less accessible form of chromatin. In contrast, hypomethylated regions are generally associated with a more open chromatin state, facilitating active gene expression [148].

### Histone modifications and chromatin accessibility

*Impact on Chromatin Accessibility* Histone modifications, such as acetylation, methylation, and phosphorylation, play a significant role in modulating chromatin structure and accessibility. Histone acetylation, for example, is typically associated with an open chromatin configuration (euchromatin) that is more accessible to transcriptional machinery. Conversely, certain histone methylations can either enhance or inhibit chromatin accessibility depending on the specific modification and its location [149]***.*** Siggens et al. [149] elucidated the pivotal roles of CHD1 and CHD2 chromatin remodeling enzymes across developmental processes, cancer biology, and cellular differentiation. Their research delineated a transcription-dependent mechanism by which CHD1 and CHD2 are recruited to transcription start sites (TSSs) of genes, as well as to intragenic and intergenic enhancer-like loci. This recruitment modulates the architecture of active chromatin regions through processes involving chromatin accessibility and the disassembly of nucleosomes. These findings underscore the critical regulatory functions of CHD1 and CHD2 in orchestrating chromatin dynamics essential for gene expression and cellular function in diverse biological contexts.

*Histone Code* The ‘histone code’ refers to the combinatorial patterns of histone modifications that regulate chromatin dynamics. For instance, trimethylation of histone H3 at lysine 27 (H3K27me3) is associated with gene silencing and a closed chromatin state [149]***.***

### Noncoding RNAs and chromatin accessibility

*Regulation of Chromatin Accessibility* Noncoding RNAs, including long noncoding RNAs (lncRNAs) and small RNAs, play crucial roles in modulating chromatin accessibility. lncRNAs can recruit chromatin-modifying complexes to specific genomic regions, altering histone modifications and DNA methylation patterns, which in turn impacts chromatin accessibility and gene expression. Jiang et al. [150] conducted a pioneering genome-wide analysis focusing on long non-coding RNAs (lncRNAs) in the Naked mole rat (NMR, *Heterocephalus glaber*), exploring their potential implications in cancer resistance. Their findings strongly indicate that lncRNAs play significant roles in the NMR's anticancer mechanisms, suggesting their crucial involvement in these processes.

*RNA-Directed DNA Methylation* Small RNAs, such as microRNAs and small interfering RNAs, can direct DNA methylation and histone modification complexes to specific target regions. This RNA-directed regulation influences chromatin accessibility and gene expression indirectly*.* Chromatin accessibility is a dynamic feature of the epigenetic landscape that integrates the effects of DNA methylation, histone modifications, and noncoding RNAs. These elements work synergistically to regulate gene expression by controlling the accessibility of DNA.

*Transcription* miRNA transcripts can live inside host gene introns, co-transcript with different genetic material, or come from independently transcribed genes. While RNA polymerase II is responsible for the transcription of most miRNAs, RNA polymerase III co-transcripts a subset of miRNAs with nearby recurring components. The first transcript, known as a pri-miRNA, consists of a hairpin loop containing a partially double-stranded area. Longer sequences can contain double-stranded regions and extend from the hairpin's 5′ and 3′ ends. [3].

**Fig. 4:**
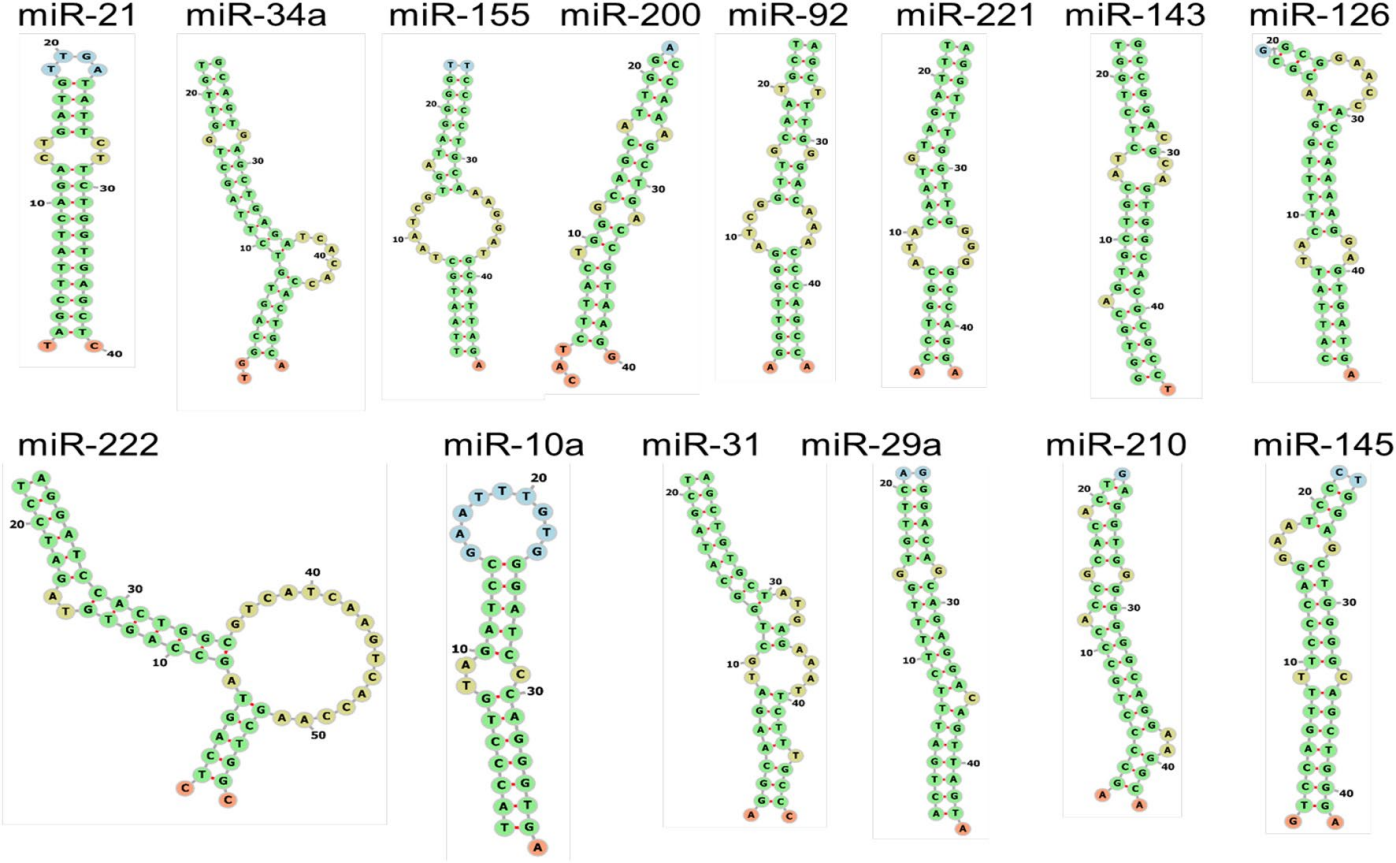
The secondary structure of microRNAs is visualized using a Force Directed Graph Layout. This figure presents the secondary structure of specific miRNAs (miR-21, miR-34a, miR-155, miR-200, miR-92, miR-221, miR-143, miR-126, miR-222, miR-10a, miR-31, miR-29a, miR-210, and miR145) as visualized through interactive graph layout software. The diagram displays the folding pattern of the miRNA molecule, highlighting key structural features such as hairpins, loops, and stems. These structural elements are crucial for miRNA function and its interaction with target mRNAs (http://mirwalk.umm.uni-heidelberg.de/human/mirna/MIMAT0000437/)

1. *DROSHA-Mediated Processing* DROSHA nuclease, in coordination with the DGCR8, performs an endoribonucleolytic cleavage at both the 5' and 3' ends of the pri-miRNA. This process generates a short hairpin, approximately 60 to 70 nucleotides in length, known as the pre-microRNA (pre-miRNA) [60, 61].
2. *Exporting from the Nucleus* The pre-miRNA associates with Exportin-5, Ran, and GTP to form a complex, aiding its passage through the nuclear pore into the cytoplasm [62].
3. *Processing by DICER1* The RISC loading complex, comprising DICER1, an Argonaute protein, and either TARBP2 or PRKRA, catches the pre-miRNA in the cytoplasm. After DICER1 cleaves the pre-miRNA, an imperfectly double-stranded miRNA with a length of 21–23 nucleotides is produced. Currently, the double-stranded miRNA has 2–3 nucleotides of protruding single-stranded 3′ ends [63].
4. *Integration with choosing strands and RISC* Within the RISC loading complex, the double-stranded miRNA is transferred to an Argonaute protein. The passenger strand is subsequently removed and degraded, while the guide strand is retained to direct the Argonaute:miRNA complex (RISC) towards target mRNAs [63, 64]. The secondary structure of micro-RNA visualization by Force Directed Graph Layout was shown in Fig. 4**.**

### RNA-mediated mechanisms in cancer: insights into regulatory processes and therapeutic implications

The human genome encodes four Argonaute proteins, particularly EIF2C1 to 4 [65]. Among these, AGO2 (EIF2C2) stands out for its capability to cleave target mRNAs with near-perfect complementarity to the guiding miRNA. While the mechanism of passenger strand removal in complexes involving other Argonautes remains incompletely understood, miRNA-loaded AGO2 complexes primarily localize at the rough endoplasmic reticulum’s cytosolic surface in association with TARBP2 or PRKRA. Additionally, TARBP2, AGO2, and DICER1 have been detected in the nucleus.

In malignancies, both pre-translational and transcriptional modifications occur within the nucleus. Examples include miR-15/16 loss linked to deletions in 13q14.3 genes in chronic lymphocytic leukemia (CLL) and mutations affecting crucial components like DGCR8, Exportin 5, Drosha, and DGCR8. In the cytoplasm of cancer cells, mutations in TRBP and Dicer impact RNA processing. Modifications in transcription or translation also affect DICER, while AGO2 is influenced by Epidermal Growth Factor Receptor-triggered phosphorylation. These alterations, affecting target mRNA through binding site mutations and 3′ UTR shortening in DICER, contribute to dysregulated cellular mechanisms specific to cancer.

Modifications in transcription or translation have an impact on the DICER. Moreover, AGO2 is impacted by Epidermal Growth Factor Receptor-triggered phosphorylation. Binding site mutations, like that found in KRAS 3′ UTR, along with 3′ UTR shortening, like in DICER, result in changes to target mRNA. Furthermore, KSRP-mediated miRNA loading to RISC facilitates selective processing. All of these alterations add to the dysregulated cellular mechanisms that are specific to cancer. The majority of those linked to the cytoplasm are post-transcriptional and involve the processing of miRNAs, while those linked to the nucleus are primarily genetic and transcriptional. The dysregulated cellular processes that are typical of cancer are caused by both kinds of changes.

Understanding the roles of non-coding RNAs (ncRNAs) and their interactions with target genes is critical for developing targeted cancer therapies. Specific miRNAs and lncRNAs have been identified with oncogenic or tumor-suppressive functions, presenting potential targets for cancer therapy [32, 33, 35, 58]. For instance, miR-34a, a well-studied tumor suppressor, has shown therapeutic potential in various cancers [66], LncRNAs, such as HOTAIR and MALAT1, are associated with cancer progression and metastasis [67]. Rupaimoole and Slack examined into microRNA-based therapies and their implications for precision medicin [3].

On the other hand, lncRNAs, known for their extensive length, participate in diverse regulatory processes involving chromatin remodeling, transcriptional control, and epigenetic modifications. They interact with DNA and histones, influencing functionality. Perturbations in these regulatory components can lead to various pathological conditions, including malignancies, neurodevelopmental anomalies, and autoimmune disorders.

### Epigenetic biomarkers for cancer diagnosis and prognosis

**Fig. 5:**
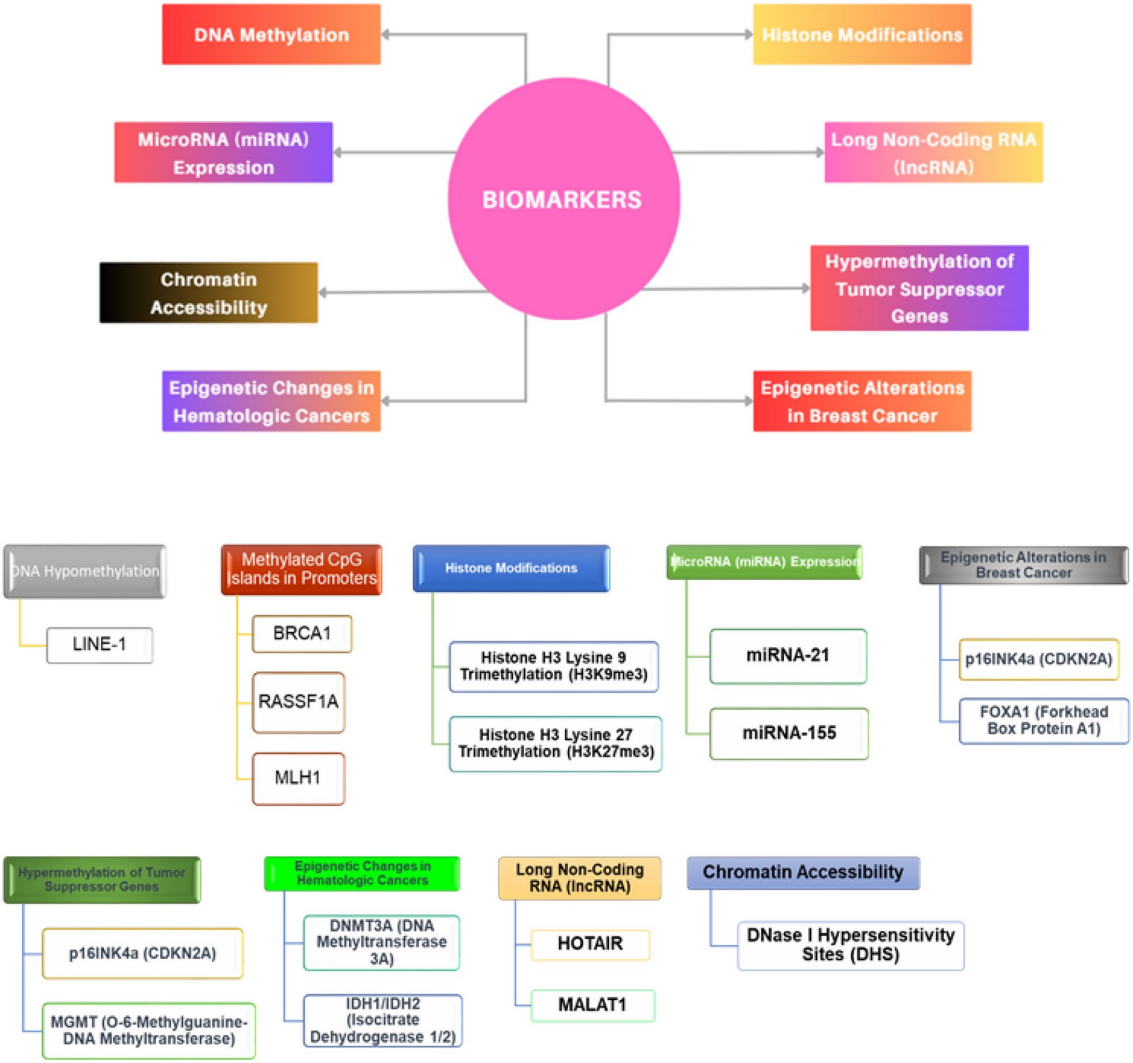
Provides a schematic representation of epigenetic biomarkers used for cancer diagnosis and prognosis. Key examples include DNA methylation, where hypermethylation of tumor suppressor genes like p16INK4a and hypomethylation of oncogenes such as MYC can signal various cancers, including melanoma and colon cancer. Histone modifications also play a crucial role; for instance, increased acetylation of histone H3 lysine 27 (H3K27ac) is linked to active gene expression in prostate cancer, while specific methylation marks like trimethylation of histone H3 lysine 4 (H3K4me3) are associated with active promoters in leukemias. Non-coding RNAs, including miR-21, are associated with poor prognosis in breast cancer and lung cancer, and HOTAIR lncRNA overexpression is linked to metastasis in breast cancer and colorectal cancer. Chromatin remodeling factors such as mutations in BRG1 (part of the SWI/SNF complex) are found in small-cell lung cancer and endometrial cancer, affecting gene expression. Additionally, genomic imprinting anomalies, such as overexpression of the IGF2 gene, are seen in Wilms’ tumor and can influence cancer progression. These biomarkers are pivotal in advancing cancer diagnosis, predicting disease outcomes, and guiding personalized treatments

Epigenetic biomarkers have become pivotal tools in cancer diagnosis, prognosis, and personalized treatment selection [85, 86]. These biomarkers unravel the intricate web of epigenetic changes in cancers like liver, stomach, and pancreatic, empowering healthcare professionals to make informed decisions. Detailed cancer epigenetic biomarkers and its types was shown in Fig. 5. Among these markers, DNA methylation patterns stand out significantly [87]. Aberrant DNA methylation at specific gene loci serves as a diagnostic hallmark for various cancers. For instance, hypermethylation of the *MLH1* gene's promoter region aids early colorectal cancer detection [88, 89].

Beyond diagnosis, epigenetic biomarkers also serve as prognostic indicators, predicting cancer progression and recurrence likelihood. They play an essential role in tailoring treatment strategies, exemplified by the predictive value of *MGMT* gene methylation status in glioblastoma response to alkylating agents like temozolomide [90]. This heralds the era of precision medicine, selecting therapies based on individual epigenetic profiles.

The integration of epigenetic agents into combination therapies effectively targets tumor cells, regulating crucial processes such as apoptosis, proliferation, migration, and combating therapy resistance. Many approaches, such as receptor-mediated endocytosis (mediated by CD44 and αv3 integrin receptors), cell-mediated homologous targeting, and TAT-mediated cell penetration, enable precise delivery of nanomedicine. Upon arrival in the tumor tissue, nanoplatforms release epigenetic drugs and other antitumor agents in response to the tumor microenvironment (TME) factors like pH, GSH, and specific enzymes, allowing for optimal synergistic effects. This combination approach shows remarkable efficacy in treating solid tumors through a variety of machinery. It combines epigenetic therapy with other antitumor treatments such as radiation, chemo, molecularly directed, traditional medicine therapy, and photoacoustic visualization. Ligands such as HA and iRGD, along with acronyms corresponding PA, PD-1, PD-L1, also receptors such as CD44 and αvβ3 integrin receptors, as well as TAT (cell-penetrating peptide), play critical roles in this integrated therapeutic approach.

Despite their immense potential, challenges persist in the widespread clinical implementation of epigenetic biomarkers. Standardized methods for detecting and interpreting these alterations are still evolving. The dynamic nature of epigenetic changes necessitates robust assays capable of capturing real-time alterations accurately. Yet, the growing body of evidence supporting their clinical utility promises enhanced cancer diagnosis, prognosis, and personalized treatment. Advances in epigenomic profiling have unveiled epigenetic biomarkers that significantly contribute to cancer diagnosis, prognosis, and treatment selection.

## Epigenetics in cancer therapy

Epigenetic treatments have emerged as a promising avenue in cancer therapy, targeting reversible modifications like DNA methylation and histone alterations [4]. These treatments encompass inhibitors for DNA methyltransferase and histone deacetylase, among others, effectively addressing the underlying molecular pathways driving cancer growth. Notable classes of epigenetic modulators include DNA methyltransferase inhibitors (like Vidaza, Dacogen, Zebularine and Guadecitabine), HDAC-inhibitors (like Zolinza, Istodax, Beleodaq, Farydak, Entinostat (MS-275), Trichostatin A (TSA), Valproic acid (VPA)), histone methyltransferase inhibitors (like EPZ-5676), and Bromodomain and extra-terminal inhibitors (like JQ1 and OTX-015) [91, 92]. Moreover, RNA interference (RNAi) therapies utilizing small interfering RNA (siRNA) and short hairpin RNA (shRNA) precisely target cancer-linked genes and reversible epigenetic modifications such as, DNA methylation and histone modifications [4, 93–95]. For instance, DNA methyltransferase inhibitors like 5-azacitidine and histone deacetylase inhibitors such as vorinostat are among the compounds developed for this purpose.

**Fig. 6:**
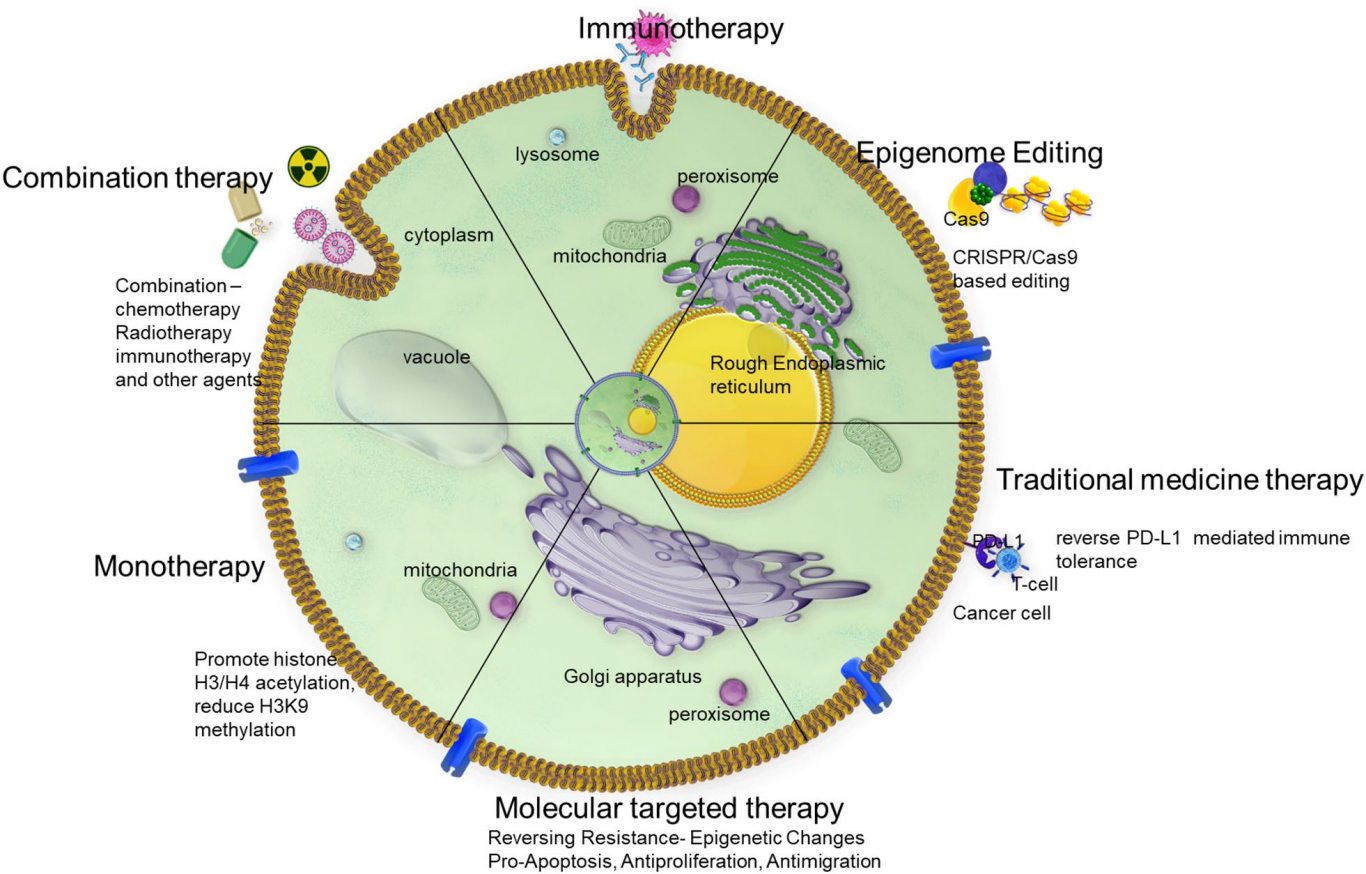
Schematic representation of various types of cancer therapies and their mechanisms of action. This figure categorizes and illustrates different cancer treatment modalities including chemotherapy, radiotherapy, targeted therapy, immunotherapy, and hormone therapy. Each therapy type is depicted with its specific mechanism of action, such as direct cytotoxic effects, DNA damage induction, targeted inhibition of cancer-specific pathways, immune system activation, and hormone receptor modulation. The diagram aims to provide a comprehensive overview of how each therapy targets cancer cells and the underlying biological mechanisms involved

A significant paradigm shift in cancer treatment involves combining epigenetic medicines with traditional therapies like immunotherapy, chemotherapy and targeted therapies (Fig. 6). This strategy can enhance treatment results, overcome drug resistance, customize treatment regimens, lessen side effects, and increase the susceptibility of cancer cells to cell death by sensitizing them to alternative therapeutic modalities. In addition to providing insightful information about the rational design of epigenetic therapies, Dawson and Kouzarides’ study summarized significant epigenetic events that contribute to the onset and progression of cancer. This work ultimately paved the way for novel strategies to fight cancer by modifying the epigenetic machinery. Sharma and Kelly’s study revealed a valuable resource for understanding the significance of epigenetics in cancer [96]. It underscores the potential of epigenetic therapies and the importance of a holistic approach that combines genetic and epigenetic insights for the development of more effective cancer treatments. This study has contributed to the growing recognition of epigenetics as a pivotal player in cancer biology and therapy.

Sigalotti et al. study underscores an epigenetic drug as immunomodulators in the treatment of solid tumors [97]. It highlights the potential of combination therapies that leverage both epigenetic modifications and immunotherapy to enhance the immune response against cancer cells. Flavahan and Gaskell's study exhibited the critical role of epigenetic plasticity in cancer biology and its impact on the hallmarks of cancer. It provides valuable insights into the complex interplay between epigenetics and cancer progression, offering a foundation for the development of innovative therapeutic strategies aimed at modulating epigenetic plasticity to improve cancer treatment outcomes [98]. Immunotherapies containing epigenetic components, such as checkpoint inhibitors like nivolumab (Opdivo) and pembrolizumab (Keytruda), may affect the tumor microenvironment's epigenetic regulation. By focusing on immunological checkpoints, these inhibitors effectively let the body’s defenses against cancer cells loose. The delicate relationship between the immune system and epigenetic mechanisms is highlighted by the interaction between immunotherapy and these processes, which presents a promising way to improve the efficacy of cancer treatment. These treatments may enhance the overall therapeutic effect by altering epigenetic variables in the tumor microenvironment, which may facilitate immune cells’ ability to identify and assault cancer cells.

### Targeted epigenetic therapies: tailoring treatment strategies

Targeted epigenetic therapies offer a personalized approach to combatting cancer by addressing specific epigenetic alterations driving disease progression. These treatments aim to restore normal gene expression patterns and impede tumorigenesis. Among these therapies are DNMTis like 5-azacitidine and decitabine, which hinder the activity of DNA methyltransferases [132]. By blocking DNA methylation, these agents reactivate silenced tumor suppressor genes, curbing cancer cell growth and promoting programmed cell death. Histone deacetylase inhibitors (HDACis) represent another crucial class of targeted epigenetic therapies. HDACis alters histone protein acetylation, influencing chromatin structure and gene accessibility. In cancers, deregulated histone deacetylation can silence critical genes. Agents like vorinostat and romidepsin reverse this process, reactivating genes that impede cancer progression [138, 140].

The integration of targeted epigenetic drugs with conventional chemotherapy or immunotherapy showcases significant promise. For instance, combining DNMTis or HDACis with conventional therapies sensitizes cancer cells to treatment, potentially enhancing therapeutic responses and countering drug resistance. Previous studies provide compelling evidence for the efficacy of these synergistic therapies in clinical settings, emphasizing their importance in improving treatment outcomes [4, 93].

### Combination therapies

Combination therapies in cancer treatment have gained considerable attention, particularly in recent years, as researchers and clinicians recognize the potential of merging epigenetic therapies with conventional treatments like chemotherapy, immunotherapy, and targeted therapies [99, 100]. Due to the complex interplay between histone deacetylation and DNA methylation in regulating gene expression, combined use of DNMT and HDAC inhibitors have been the subject of much research. High methylation regions of DNA are typically associated with densely packed chromatin topologies characterized by under-acetylated histone lysines. At the moment, combining DNMT inhibitors like azacitidine, decitabine, and hydralazine with HDAC inhibitors like pracinostat, valproic acid, entinostat, and vorinostat is showing encouraging early efficacy. Thorough clinical testing is being done on this method for hematological malignancies as well as solid tumors [99].

- 1.* Enhanced Efficacy* Combining epigenetic therapies with traditional treatments can enhance the overall effectiveness of cancer therapies. Epigenetic modifiers, such as DNMTis and HDACis, can sensitize cancer cells to the cytotoxic effects of chemotherapy drugs. This synergistic effect may result in increased cancer cell death and tumor regression.
- 2.* Overcoming Drug Resistance* Drug resistance remains a major challenge in cancer treatment. Epigenetic changes are often implicated in the development of resistance to chemotherapy and targeted therapies. By targeting epigenetic alterations, combination therapies can disrupt the mechanisms that cancer cells use to evade treatment, potentially re-sensitizing them to the effects of these therapies.
- 3. *Personalized Treatment* The combination of epigenetic therapies with other treatments allows for a more personalized approach to cancer care. Epigenetic biomarkers can help identify patients who are most likely to benefit from these combination approaches. This personalized treatment strategy optimizes therapeutic efficacy while reducing unnecessary side effects for individuals who may not respond as favorably to conventional treatments alone.
- 4. *Minimized Side Effects* Conventional cancer treatments like chemotherapy often come with significant side effects due to their cytotoxic nature. Combining them with epigenetic therapies can allow for lower doses of cytotoxic agents while maintaining or even enhancing treatment efficacy. This reduction in side effects can significantly improve the quality of life for cancer patients.
- 5. *Potential for Immunotherapy Synergy* By using the immune system to attack cancer cells, vaccinations have completely changed the way that cancer is treated. Combining epigenetic therapies using immunotherapy may further enhance the immune system’s ability to recognize and attack cancer cells. Epigenetic modifications can influence the tumor microenvironment, making it more immune-responsive and potentially increasing the effectiveness of immunotherapies.

The integration of epigenetic therapies with traditional cancer treatments represents a promising avenue in the ongoing quest for more effective cancer therapies. As research in this field continues to evolve, clinicians are likely to increasingly adopt combination approaches that capitalize on the synergistic effects of epigenetic modifiers and conventional treatments, ultimately leading to improved patient outcomes in the battle against cancer.

## Advances in oligonucleotide-based therapies: ASOs and AntimiRs

### ASOs: enhancing target specificity and stability

The evolution of ASOs began with the introduction of phosphorothioate nucleotides, a first-generation modification involving the substitution of non-bridging oxygen in the phosphate group with sulphur [60, 87, 91]. This alteration bolstered ASO stability within cellular environments, rendering internucleotide linkages resistant to nucleases degradation. Notably, this modification retained RNase H activation, crucial for mRNA target cleavage and subsequent suppression of target gene expression. Expanding on this basis, phosphorothioate nucleotides with 2′–O-methyl groups showed increased binding attraction for specific messenger RNA, markedly increased nuclease obstruction, and increased in vivo stability [60, 87, 91, 101].

### Expanding target delivery in therapeutic strategies

In recent breakthroughs, a peptide backbone modification of nucleic acids has been developed to enhance tumor delivery. This innovation involves the use of a pH low insertion peptide (pHLIP)-modified antimiR [102], where the peptide antisense nucleotide is modified through the addition of pHLIP via a disulfide bond. This conjugation enables efficient entry into cancer cells, capitalizing on the low pH in the tumor microenvironment. Under such conditions, pHLIP undergoes a pH-dependent conformational change, facilitating cargo release within the cytosol [102–104]. To facilitate the delivery of peptide nucleic acids (PNAs) into cells, it is necessary to attach the PNA to a larger molecule, commonly a cell-penetrating peptide or nanoparticle [105].

Clausse et al. [105] conducted a study focusing on a PNA-derived compound subject to structural modification through the incorporation of cyclic tetrahydrofuran (THF) moieties. This modification demonstrated enhanced cellular permeability under specific conditions. Additionally, the researchers engineered a variant, referred to as thyclotides, in which THF substitutions were introduced at each oligomeric position. Thyclotides were strategically designed to target miR-21, a distinct form of genetic material. The modified thyclotides exhibited superior cellular uptake compared to unaltered PNA molecules utilizing an aminoethylglycine backbone. Moreover, an optimized thyclotide variant demonstrated autonomous cellular entry, obviating the need for supplementary cell-penetrating peptides. This specialized iteration proficiently engaged with miR-21, resulting in downregulation of miR-21 expression and concurrent upregulation of three downstream targets within the cellular milieu, namely *PTEN, Cdc25a,* and *KRIT1*.

### Targeting mature miRNAs: OncomiRs and AntimiRs

AntimiRs share structural similarities with ASOs but are designed to bind directly to the mature strand of the targeted miRNA, instigating a functional blockade [106]. Recent studies have explored modifications of antimiRs previously developed for ASOs [60, 106, 107]. An antimiR that was engineered to target miR-122 with a 2-O-methoxyethyl modification, for instance, demonstrated better target modulation than antimiRs that did not have this particular modification. Noteworthy advancements include Locked Nucleic Acid modified antimiRs, which mimic a ribonucleotide’s C3′-endo conformation [108]. LNA mixmers, featuring repeated patterns of deoxyribonucleotides and locked ribonucleotides, demonstrated promising results in various in vivo models.

OncomiRs, microRNAs associated with cancer progression, present promising targets for therapeutic intervention using antimiRs. For instance, miR-10b is implicated in various solid tumors like breast and glioma, regulating genes *NF1, CDH1, E2F1, PIK3CA, ZEB1,* and *HOXD10* [78]. Orthotopic administration of locked nucleic acid antimiRs against miR-10b has shown promise in glioblastoma and breast cancer models. Another notable oncomiR, miR-155, is overexpressed in a range of solid tumors including liver, lung, kidney, glioma, and pancreas, as well as in B cell lymphomas and lymphoid leukemias. Its effects are mediated through targeting genes like *SHIP, SPI1, HDAC4, RHOA, SOCS1, BCL2, JMJD1A, SOX6, SMAD2, SMAD5,* and *TP53INP1*. Experimental approaches using miR-155-overexpressing models have demonstrated the potential of antimiRs as therapeutic agents, particularly in lymphomas. Additionally, pHLIP-conjugated antimiRs offer a targeted delivery mechanism for inhibiting miR-155. Moreover, miR-221 and miR-222 play roles in solid tumors such as liver, pancreas, and lung cancers, targeting genes *CDKN1B, CDKN1C, BMF, RB1, WEE1, APAF1, ANXA1, and CTCF*. In liver cancer models, cholesterol-conjugated antimiRs against miR-221 and miR-222 have shown promise in targeting HCC xenografts. These findings collectively underscore the potential of antimiRs as a targeted therapeutic approach in mitigating the impact of dysregulated oncomiRs in cancer.

### Epigenetic editing and precision medicine

CRISPR/Cas9 screening in oncology plays a pivotal role in identifying genotype-specific vulnerabilities. Through targeted gene deletion, the viability of cancer cells can be selectively diminished, offering a potential avenue for discovering therapeutic targets [109, 110]. Another crucial application involves elucidating genes that either collaborate with a drug or develop resistance against it. The combination of CRISPR screening with drug perturbation provides invaluable insights into by what means cancer responds to treatment [111]. Stadtmauer et al. reported that, three patients with refractory cancer are the subjects of a first-ever phase 1 clinical trial designed to assess the safety and feasibility of multiplex CRISPR-Cas9 editing in reprogramming T cells. *TCRα* (TRAC) and *TCRβ* (TRBC), the two genes that encode the native T cell receptor (TCR) chains, were purposefully taken out of the T cells in this intervention. This strategic deletion served the purpose of mitigating TCR mispairing and amplifying the expression of a synthetic TCR transgene, specialized for targeting the NY-ESO-1 cancer antigen. In addition, a 3rd gene that codes for the programmed cell death protein 1 (PD-1; PDCD1) was deleted to improve the immune response against tumors. After the patients were given engineered T cells, all three of the targeted genomic loci successfully underwent long-lasting engraftment. Chromosome translocations were seen, but over time, their frequency gradually decreased [112]. In order to validate and assess acute myeloid leukemia -enriched dependencies in vivo, Lin et al. developed a CRISPR screening strategy that uses orthotopic xenograft models. This approach includes CRISPR-competent AML patient-derived xenograft (PDX) mice that are tractable for genome editing [110]. SLC5A3 has been discovered by Lin et al. [110] as a metabolic vulnerability in AML, while MARCH5 is a crucial regulator of apoptosis. These findings suggest new avenues for therapeutic intervention [110].

Through the application of genome-wide CRISPR/Cas9 library screening, Huang et al. have identified *DUSP4* as the crucial gene associated with the emergence of resistance to Lenvatinib in hepatocellular carcinoma (HCC). This discovery offers a substantial breakthrough in addressing resistance to tyrosine kinase inhibitors. The deficiency of *DUSP4* leads to the development of resistance to Lenvatinib by reactivating the functions of ERK and MEK in HCC patients undergoing Lenvatinib treatment [113]. In the clinical management of lung cancer and other malignancies, the use of receptor tyrosine kinase (RTK)/Ras/mitogen-activated protein kinase (MAPK) pathway inhibitors has shown promise. However, a significant number of patients still exhibit suboptimal responses to these treatments. Notably, CRISPR/Cas9-mediated gene deletion screening in lung cancer cells revealed that the absence of the *KEAP1* gene, in conjunction with multiple targeted RTK/Ras/MAPK pathway inhibitors, induces metabolic adaptations. This enables cells to proliferate even in the absence of requisite MAPK signaling. This loss-of-function screening method proves instrumental in assessing the effectiveness of related drugs in clinical trials and aids in making informed treatment choices.

In the realm of carcinogenesis, somatic mutations yield neoantigens that elicit a potent T-cell response. However, these mutations can also confer resistance to immunotherapeutic interventions. Cancer cells transduced with sgRNA libraries were cultured alongside cells from the immune system to obtain a better understanding of how cancer cells become resistant to attacks by immune systems. The identification of sgRNAs displaying enrichment or depletion patterns in the population of cancer cells that survived was made possible by later NGS. This methodological approach aids in the delineation of genetic perturbations that dictate the resistance or sensitivity of cancer cells to immune cell-mediated cytotoxicity [114].

### Nanotechnology/nano-medicine: revolutionizing cancer treatment

Nanomedicine has revolutionized cancer treatment by leveraging nanoparticles (NPs), typically within the size range of 1 to 100 nm, for various therapeutic and diagnostic purposes. NPs-based epigenetic drug delivery, offering a precision-oriented strategy to target the underlying molecular mechanisms driving tumorigenesis [115]. One of its key advantages lies in targeted drug delivery, where NPs can be precisely engineered to target cancer cells, sparing healthy tissues and minimizing the often debilitating side effects associated with traditional cancer treatments [40, 43]. Moreover, nanomedicine has addressed the issue of poor drug solubility, a common challenge in anticancer therapies. NPs can encapsulate these drugs, improving their solubility and overall bioavailability, thus enhancing their effectiveness [41, 42, 116, 117]. By doing so, it overcomes several challenges associated with conventional epigenetic therapies, such as off-target effects and systemic toxicity. These NPs act as specialized vehicles, ferrying epigenetic drugs directly to the cancer cells while sparing healthy tissues. This is particularly crucial in the context of epigenetic modifications, which can exert wide-ranging effects on cellular behavior. The tailored delivery ensures that the epigenetic agents reach their intended targets, where they can elicit specific changes in gene expression patterns, chromatin structure, and other epigenetic features. Table 3 provides an overview of the key challenges faced when using nanomaterials in cancer therapy and suggests potential solutions to address these issues. Additionally, the summary of research on nanomaterials and their impact on epigenetic changes in cancer treatment is shown in Table 4.

**Table 3:** The key challenges and potential solutions associated with using nanomaterials in cancer therapy

Challenge	Description	Potential solutions	References
Biocompatibility and Toxicity	Nanomaterials may induce adverse effects or toxicity in healthy tissues	Develop biocompatible coatings and surface modifications Conduct thorough in vivo and in vitro testing	[161]
Targeted delivery	Achieving precise delivery of nanomaterials to tumor cells while avoiding healthy tissues is challenging	Utilize targeted ligands (e.g., antibodies, peptides) to improve specificity Employ stimuli-responsive materials	[162, 163]
Drug resistance	Tumors may develop resistance to the therapeutic agents delivered by nanomaterials	Design multi-drug delivery systems to overcome resistance Incorporate combination therapies	[164, 165]
Scale-Up and Manufacturing	Scaling up production from laboratory to clinical-grade materials can be complex and costly	Optimize synthesis and purification processes Develop standardized protocols for large-scale production	[166, 167]
Regulatory and safety Issues	Navigating the regulatory landscape and ensuring safety for clinical use can be difficult	Adhere to regulatory guidelines and conduct extensive safety evaluations Engage with regulatory agencies early in the development process	[168, 169]
Stability and shelf-life	Nanomaterials may have limited stability and short shelf-life, impacting their effectiveness and usability	Develop stable formulations and storage conditions Implement encapsulation techniques to enhance stability	[170, 171]
Biological clearance and accumulation	Nanomaterials can accumulate in non-target organs, leading to potential toxicity	Engineer nanoparticles for controlled release and enhanced clearance Use biodegradable materials to minimize accumulation	[172, 173]
Cost of production	The cost of developing and producing nanomaterials can be high	Explore cost-effective production methods Collaborate with industry partners to share costs and resources	[73, 174]
Ethical and social implications	There may be concerns about the ethical use and societal impact of advanced nanotechnologies in medicine	Engage in public dialogue and address ethical considerations Ensure transparency in research and development processes	[175]
Limited clinical success	Some nanomaterial-based therapies may not show expected clinical outcomes	Conduct robust clinical trials and longitudinal studies Refine nanomaterial designs based on clinical feedback	[176]

**Table 4:** Summary of research on nanomaterials and their impact on epigenetic changes in cancer treatment

S. No	Nanomaterial type	Description	Impact on epigenetic changes	Examples/references
1	Gold nanoparticles	Spherical gold particles with size ranging from 1 to 100 nm	Can influence gene expression by modifying DNA methylation and histone acetylation	Enhances expression of tumor suppressor genes in breast cancer [177]
2	Silver nanoparticles	Nanoscale silver particles with antibacterial properties	Induces DNA damage and alters histone modifications, potentially reactivating silenced genes	Modulates histone H3 acetylation in lung cancer cells [178]
3	Magnetic nanoparticles	Iron oxide particles used for imaging and targeted therapy	Can affect gene expression by altering histone marks and DNA methylation patterns	Reverses aberrant gene silencing in prostate cancer [179]
4	Quantum dots	Semiconductor nanoparticles with unique optical properties	Influences epigenetic regulators and enhances gene delivery systems	Improves delivery of epigenetic drugs in colon cancer [180]
5	Carbon nanotubes	Cylindrical nanostructures with high mechanical strength and conductivity	Can affect histone modifications and gene expression through cellular uptake	Alters gene expression and histone acetylation in breast cancer cells [181]
6	Liposomes	Nanoparticles with a lipid bilayer used for drug delivery	Can modulate epigenetic pathways by delivering small molecules or RNA molecules	Enhances gene silencing in ovarian cancer through RNA interference [182]
7	Polymeric nanoparticles	Nanoparticles made from biodegradable polymers	Can modify epigenetic landscapes by delivering gene-specific inhibitors or activators	Targets specific gene expression in glioblastoma [183]
8	Dendrimers	Branched nanomaterials with a high degree of functionalization	Influences gene expression by targeting epigenetic modulators and gene silencing	Facilitates gene therapy by modifying histone acetylation in liver cancer [184]
9	Mesoporous silica nanoparticles	Nanoparticles with a porous structure for drug delivery and imaging	Can affect epigenetic modifications by controlled release of epigenetic drugs	Enhances the efficacy of epigenetic drugs in pancreatic cancer [185]

*nm* nanometer, *DNA* Deoxyribonucleic acid, *RNA* Ribonucleic acid

Another significant benefit of nanomedicine is the ability of NPs to sustain drug release over time, offering a prolonged therapeutic effect and reducing the need for frequent dosing. These NPs can also serve as valuable tools in cancer imaging and diagnosis, acting as contrast agents in techniques like MRI or PET scans. This aids in early cancer detection and monitoring treatment response. Sendi et al. has reported the development of a miR-122 nanoformulation as a therapeutic agent to prevent liver metastases [118]. They constructed a miR-122 lipid calcium phosphate (Gal-LCP) nanoformulation that is specifically targeted at galactose. This nanotherapeutic selectively and highly efficiently delivered miR-122 into hepatocytes without causing any significant toxicity, thisstudy describes a method for enhancing miRNA delivery through nanomedicine to improve cancer prevention and treatment.

Furthermore, the concept of theranostics has emerged, wherein certain NPs can perform dual roles as both diagnostic and therapeutic agents. They can be employed not only to diagnose cancer but also to deliver treatment simultaneously, offering a more comprehensive and targeted approach to patient care. In the fight against drug resistance, nanomedicine strategies have also proven instrumental.

By altering drug pharmacokinetics and bypassing resistance mechanisms, NPs can help overcome drug resistance in cancer, providing new hope for patients facing this formidable challenge. Overall, nanomedicine has ushered in a promising era in cancer research and treatment, offering innovative solutions to enhance efficacy, reduce side effects, and improve patient outcomes.

- Lutetium-177 PSMA (Lu-177 PSMA): Used in the treatment of metastatic castration-resistant prostate cancer, Lu-177 PSMA is a radiopharmaceutical that targets prostate-specific membrane antigen (PSMA) expressing cells [119].
- Gallium-68 PSMA (Ga-68 PSMA): This radiopharmaceutical is used for PET imaging in prostate cancer patients to detect the presence and spread of PSMA-expressing tumors [120].
- Yttrium-90 Ibritumomab Tiuxetan (Zevalin): Zevalin is used for the treatment of certain types of non-Hodgkin's lymphoma. It combines a monoclonal antibody targeting CD20 with a radioactive isotope [121].
- Iodine-131 Metaiodobenzylguanidine (MIBG): Used in the treatment of neuroblastoma, MIBG is a radiopharmaceutical that targets neuroblastoma cells [122].
- Radium-223 Dichloride (Xofigo): Xofigo is used for the treatment of metastatic castration-resistant prostate cancer. It is a targeted alpha-particle emitting radiopharmaceutical [123].
- Iodine-131 I-Tositumomab (Bexxar): Bexxar was used for the treatment of certain types of non-Hodgkin's lymphoma. It combined a monoclonal antibody targeting CD20 with a radioactive isotope [124].
- Nanoparticle Albumin-Bound Paclitaxel (Abraxane): While not strictly a theranostic drug, Abraxane is an example of a nanoparticle-based drug delivery system. It delivers paclitaxel to cancer cells more efficiently than traditional formulations [125].
- ^64^Cu-DOTATATE (Ga-DOTATATE): This radiopharmaceutical is used for imaging and therapy in neuroendocrine tumors expressing somatostatin receptors [126].
- ^131^I-Metaiodobenzylguanidine (131I-MIBG): Similar to MIBG, this radiopharmaceutical is used in the treatment of neuroblastoma [127].
- Rhenium-186 Liposomes (Re-186 Liposomes): Liposome-based drug delivery systems loaded with the beta-emitting isotope rhenium-186 have shown potential in cancer therapy [128].
- ^177^Lu-DOTATATE (Lutathera): Lutathera is used in the treatment of neuroendocrine tumors expressing somatostatin receptors [129].

#### Nanoparticle-based therapies: enhancing efficacy and selectivity

The effectiveness of NPs -based epigenetic medication delivery is its capacity to capitalize on the tumor vasculature’s increased permeability and retention (EPR) effect. This condition causes leaky blood arteries and poor lymphatic drainage, which is characteristic of many solid tumors and allows nanoparticles to collect specifically in the tumor microenvironment. These nanoparticles can also be modified on the surface and fitted with ligands to enable active targeting, which increases their specificity for cancer cells [130, 131]. For instance, NPs was used to deliver fulvestrant together with abemaciclib, a powerful inhibitor of cyclin-dependent kinases 4 and 6 (CDK4/6). This combination was intended to prevent the emergence of drug resistance, which is frequently linked to extended use of fulvestrant. Targeting peptides on the NPs surface enabled targeted drug release, guaranteeing targeted toxicity in tumor tissues while protecting healthy tissue. The NP formulation, known as PPFA-cRGD, showed no discernible side effects in mouse or Bama tiny pig models while effectively eliminating tumor cells in breast cancer organoid and orthotopic models [131]. Ligands like hyaluronic acid (HA) and iRGD can be utilized to exploit overexpressed receptors on the surface of cancer cells, ensuring a higher affinity for the target site. This precision-focused approach not only maximizes the therapeutic effect but also minimizes the exposure of healthy tissues to the epigenetic agents, mitigating potential side effects. Moreover, the NPs carriers can be designed to respond to specific stimuli within the tumor microenvironment, such as pH levels, concentrations of glutathione (GSH), or enzymatic activity. This allows for controlled release of the epigenetic drugs, NPs and the tumor microenvironment exemplifies the sophistication and adaptability of this approach. Reda et al. presented antigen release agent and checkpoint inhibitor (ARAC), a NPs-based immunotherapy. This novel strategy was created specially to increase the potency of cell death protein ligand 1 (PD-L1) inhibitors. ARAC functions by delivering a programmed PD-L1 antibody inside a nanoparticle framework along with a polo-likekinase1 (PLK1) inhibitor (volasertib) [130].

Furthermore, the integration of epigenetic therapy with NPs-based delivery systems holds immense promise in overcoming drug resistance, a pervasive challenge in cancer treatment (Fig. 6). NPs can encapsulate a combination of epigenetic drugs, each targeting different aspects of the epigenetic landscape. Studies have shown that encapsulating Azacitidine in NPs offers a means of targeted drug delivery, which can improve its bioavailability and therapeutic impact across various cancer types [132, 133]. This multifaceted approach can disrupt the adaptive strategies employed by cancer cells to evade treatment, effectively restoring their sensitivity to therapeutic interventions. Additionally, NPs-based delivery systems open avenues for combination therapies, where epigenetic agents can be synergistically paired with other anti-cancer treatments. For example, NPs can be engineered to co-deliver chemotherapeutic drugs or molecularly targeted therapies alongside epigenetic agents, unleashing a potent and multifaceted assault on cancer cells. This combinatorial approach addresses the heterogeneity of cancer, targeting multiple vulnerabilities simultaneously and significantly improving treatment outcomes. In essence, NPs-based epigenetic drug delivery represents a paradigm shift in cancer therapy, offering a refined and tailored strategy to combat this complex and dynamic disease. Elzayat et al. developed GEF-AZT-NLC, a formulation of Nanostructured Lipid Carrier (NLC) designed to treat metastatic-resistant lung cancer, by combining Gefitinib with Azacitatidine [132]. Simlilarly, 4-(N)-stearoyl gemcitabine (GemC18), a fatty-acid amide prodrug of the nucleoside analog gemcitabine, was encapsulated using poly (lactic-co-glycolic acid) (PLGA) NPs or microspheres. The most effective anti-tumor impact was shown by the depot formulation, which used PLGA NPs loaded with GemC18. This effect may have been caused by the injection site's regulated release of GemC18 [134]. Guadecitabine, as a next-generation DNMTi, has garnered interest for its potential to overcome limitations associated with earlier inhibitors [135].

Ruan et al. developed a dual bioresponsive gel depot that can respond to the tumor microenvironment's (TME) acidic pH and reactive oxygen species (ROS). This depot enables the simultaneous delivery of both anti-PD1 antibody (aPD1) and Zebularine [136], an HMA. Additionally, their research shows that this combination therapy promotes cancer cells’ immunegenicity and aids in the reversal of the tumor microenvironment's immunosuppressive effects [137]. The investigation of NPs-based formulations for SGI-110, a second-generation DNMTi developed by Astex pharmaceuticals, represents an exciting development in the field. These studies suggest that harnessing the potential of nanomedicine in conjunction with DNMTis holds significant promise for advancing the efficacy and precision of epigenetic-based cancer therapies, ultimately offering hope for improved outcomes for patients with several forms of cancer [132–135, 137]. Vorinostat, a Histone Deacetylase (HDAC) inhibitors, for instance, has been extensively studied in nanoparticle-based formulations [138, 139], demonstrating potential for enhanced drug delivery and efficacy against various cancer types. The combined use of the topoisomerase II inhibitor etoposide (ETOP) with the histone deacetylase inhibitor vorinostat (VOR) was studied by Kumar et al. [139].

Human cervical cancer cells (HeLa) showed a synergistic benefit from this concurrent treatment method. Moreover, compared to the administration of the free medicines, these combination treatment medications (VOR and ETOP) showed an even more strong synergistic cytotoxic impact when they were encapsulated in poly(ethylene glycol) monomethacrylate (POEOMA) nanogels. The enhanced caspase 3/7-mediated apoptosis was found to be the cause of the drug-loaded POEOMA nanogels’ improved synergistic cell killing efficacy [139]. Romidepsin, another HDAC inhibitor, has shown promise when explored in nanoparticle formulations, suggesting improved therapeutic outcomes. Likewise, investigations into nanoparticle-based delivery systems for Belinostat have highlighted the potential for targeted therapy in cancer treatment. For the targeted treatment of diffuse intrinsic pontine glioma (DIPG) with p53-induced protein phosphatase 1 (PPM1D) mutation, Shan et al. have created a nano drug delivery system that uses functionalized macrophage exosomes loaded with PPM1D-siRNA and panobinostat. When compared to giving the medications in their free form, this method shows higher drug delivery efficiency and more powerful therapeutic benefits [140]. Natural HDAC inhibitor Trichostatin A has been the subject of research exploring its encapsulation in nanoparticles, offering a means of targeted drug delivery [27]. Additionally, NPs-based formulations of Entinostat have been under investigation, showing potential for improved drug delivery and therapeutic efficacy [141]. These studies collectively demonstrate the exciting potential of combining HDAC inhibitors with nanomedicine strategies, offering a path toward more effective and precise epigenetic-based cancer therapies, which could significantly impact the treatment landscape for various types of cancer.

Small interfering RNA (siRNA) is a class of RNA molecules that can specifically target and silence the expression of particular genes. In the realm of nanomedicine, researchers have harnessed the potential of siRNA by encapsulating or conjugating it with nanoparticles. This approach enhances the stability, delivery precision, and targeting capability of siRNA, enabling more effective and precise gene silencing for therapeutic purposes. Several notable examples of siRNA-based nanomedicine drugs and formulations have been investigated or developed [140–143]. Patisiran, an FDA-approved RNA interference (RNAi) therapeutic, addresses polyneuropathy in adults with hereditary transthyretin-mediated amyloidosis (hATTR) using lipid nanoparticles for the delivery of siRNA targeting the transthyretin Strosberg, Caplin gene [129]. Similarly, Givosiran, another FDA-approved RNAi therapeutic, combats acute hepatic porphyria by employing lipid nanoparticles for siRNA delivery, specifically targeting aminolevulinate synthase 1 (ALAS1) mRNA in the liver [144].

## Conclusion

In conclusion, the intricate interplay between genetic mutations and epigenetic alterations underscores the pivotal role of epigenetics in cancer research and treatment. The review has underscored the significance of DNA methylation, histone modifications, and non-coding RNAs, particularly microRNAs and long non-coding RNAs, as key regulators of cancer-related gene expression. By exploring the potential of epigenetic-based therapies, including oligonucleotide-based strategies such as antisense oligonucleotides and antimiRs, this article has illuminated the promising landscape of selectively modulating specific epigenetic markers involved in tumorigenesis. Furthermore, the concept of epigenetic editing has been discussed, offering a glimpse into the potential future of precision medicine for cancer patients. The exploration of epigenetic biomarkers for early cancer detection and prognosis, coupled with the integration of nanomedicine into cancer therapy, presents innovative approaches poised to enhance therapeutic efficacy. These advancements collectively drive the field of precision oncology forward, promising improved patient outcomes in the relentless fight against cancer. The continuous evolution and integration of these epigenetic-based strategies hold great promise for revolutionizing cancer prevention, diagnosis, and treatment, ultimately offering hope for better outcomes and quality of life for patients.

## Data Availability

No datasets were generated or analysed during the current study.

## References

[1] Cusenza VY, et al. The lncRNA epigenetics: the significance of m6A and m5C lncRNA modifications in cancer. Front Oncol. 2023;13:1063636. 10.3389/fonc.2023.1063636. 36969033 10.3389/fonc.2023.1063636 PMC10033960

[2] Nencioni A, et al. Fasting and cancer: molecular mechanisms and clinical application. Nat Rev Cancer. 2018;18(11):707–19. 10.1038/s41568-018-0061-0. 30327499 10.1038/s41568-018-0061-0 PMC6938162

[3] Rupaimoole R, Slack FJ. MicroRNA therapeutics: towards a new era for the management of cancer and other diseases. Nat Rev Drug Discov. 2017;16(3):203–22. 10.1038/nrd.2016.246. 28209991 10.1038/nrd.2016.246

[4] Zhang J, et al. Emerging epigenetic-based nanotechnology for cancer therapy: modulating the tumor microenvironment. Adv Sci. 2023;10(7): e2206169. 10.1002/advs.202206169. 10.1002/advs.202206169 PMC9982594 36599655

[5] Janin M, Davalos V, Esteller M. Cancer metastasis under the magnifying glass of epigenetics and epitranscriptomics. Cancer Metastasis Rev. 2023. 10.1007/s10555-023-10120-3. 37369946 10.1007/s10555-023-10120-3 PMC10713773

[6] Chu DT, Ngo AD, Wu CC. Epigenetics in cancer development, diagnosis and therapy. Prog Mol Biol Transl Sci. 2023;198:73–92. 10.1016/bs.pmbts.2023.01.009. 37225325 10.1016/bs.pmbts.2023.01.009

[7] Feinberg AP, Levchenko A. Epigenetics as a mediator of plasticity in cancer. Science. 2023;379(6632):eaaw3835. 10.1126/science.aaw3835. 36758093 10.1126/science.aaw3835 PMC10249049

[8] Pihl C, et al. Improving the efficacy of photodynamic therapy for actinic keratosis: a comprehensive review of pharmacological pretreatment strategies. Photodiagnosis Photodyn Ther. 2023;43:103703. 10.1016/j.pdpdt.2023.103703. 37429460 10.1016/j.pdpdt.2023.103703

[9] Krishnamoorthy K, et al. Improved specificity for breast cancer screening using an oncogenic (miRNA-21) and a gene suppressor (miRNA-195) miRNA in the serum for a point of care (POC) screening solution. Biomed Environ Sci. 2023;36(6):549–52. 10.3967/bes2023.067. 37424249 10.3967/bes2023.067

[10] Zhang H, et al. LncRNA MCM3AP-AS1 enhances cell invasion, migration and tumor formation in non-small cell lung cancer cells by epigenetic inhibition of miR-34a. Curr Pharm Biotechnol. 2023. 10.2174/1389201024666230223113047. 36815649 10.2174/1389201024666230223113047

[11] Weir HK, et al. Heart disease and cancer deaths—trends and projections in the United States, 1969–2020. Prev Chronic Dis. 2016;13:E157. 10.5888/pcd13.160211. 27854420 10.5888/pcd13.160211 PMC5127176

[12] Cao W, et al. Changing profiles of cancer burden worldwide and in China: a secondary analysis of the global cancer statistics 2020. Chin Med J. 2021;134(7):783–91. 10.1097/CM9.0000000000001474. 33734139 10.1097/CM9.0000000000001474 PMC8104205

[13] Ferlay J, et al. Cancer statistics for the year 2020: an overview. Int J Cancer. 2021. 10.1002/ijc.33588. 33818764 10.1002/ijc.33588

[14] Imamura J, et al. Lineage plasticity and treatment resistance in prostate cancer: the intersection of genetics, epigenetics, and evolution. Front Endocrinol. 2023;14:1191311. 10.3389/fendo.2023.1191311. 10.3389/fendo.2023.1191311 PMC10349394 37455903

[15] Singh VK, Kainat KM, Sharma PK. Crosstalk between epigenetics and tumor promoting androgen signaling in prostate cancer. Vitam Horm. 2023;122:253–82. 10.1016/bs.vh.2022.11.007. 36863797 10.1016/bs.vh.2022.11.007

[16] Szczepanek J, et al. Harnessing epigenetics for breast cancer therapy: the role of DNA methylation, histone modifications, and MicroRNA. Int J Mol Sci. 2023. 10.3390/ijms24087235. 37108398 10.3390/ijms24087235 PMC10138995

[17] Arif KMT, et al. Regulatory mechanisms of epigenetic miRNA relationships in human cancer and potential as therapeutic targets. Cancers. 2020. 10.3390/cancers12102922. 33050637 10.3390/cancers12102922 PMC7600069

[18] Anchimowicz J, Wyzewski Z, Switlik W. Role of the glucosinolates in cancer epigenetics. Postepy Biochem. 2023;69(2):96–103. 10.18388/pb.2023_478. 37493557 10.18388/pb.2023_478

[19] Bu Z, et al. Sequential ubiquitination and phosphorylation epigenetics reshaping by MG132-loaded Fe-MOF disarms treatment resistance to repulse metastatic colorectal cancer. Adv Sci. 2023;10(23): e2301638. 10.1002/advs.202301638. 10.1002/advs.202301638 PMC10427397 37303273

[20] Clermont PL. Epigenetics-based diagnostic and therapeutic strategies: shifting the paradigm in prostate cancer. Epigenomics. 2023;15(2):75–87. 10.2217/epi-2023-0045. 36974615 10.2217/epi-2023-0045

[21] Ragavi R, et al. Epigenetics regulation of prostate cancer: biomarker and therapeutic potential. Urol Oncol. 2023;41(8):340–53. 10.1016/j.urolonc.2023.03.005. 37032230 10.1016/j.urolonc.2023.03.005

[22] Tran TO, Lam LHT, Le NQK. Hyper-methylation of ABCG1 as an epigenetics biomarker in non-small cell lung cancer. Funct Integr Genom. 2023;23(3):256. 10.1007/s10142-023-01185-y. 10.1007/s10142-023-01185-y 37523012

[23] Wang T, et al. Lactate-induced protein lactylation: a bridge between epigenetics and metabolic reprogramming in cancer. Cell Prolif. 2023;56(10): e13478. 10.1111/cpr.13478. 37060186 10.1111/cpr.13478 PMC10542650

[24] Chen CY, et al. Significance of hypermethylation of tumor-suppressor genes PTGER4 and ZNF43 at CpG sites in the prognosis of colorectal cancer. Int J Mol Sci. 2022. 10.3390/ijms231810225. 36142151 10.3390/ijms231810225 PMC9499344

[25] Kulis M, Esteller M. DNA methylation and cancer. Adv Genet. 2010;70:27–56. 10.1016/B978-0-12-380866-0.60002-2. 20920744 10.1016/B978-0-12-380866-0.60002-2

[26] Li W, Xu L. Epigenetic function of TET Family, 5-methylcytosine, and 5-hydroxymethylcytosine in hematologic malignancies. Oncol Res Treat. 2019;42(6):309–18. 10.1159/000498947. 31055566 10.1159/000498947

[27] Tsai HC, Baylin SB. Cancer epigenetics: linking basic biology to clinical medicine. Cell Res. 2011;21(3):502–17. 10.1038/cr.2011.24. 21321605 10.1038/cr.2011.24 PMC3193419

[28] Shinjo K, Kondo Y. Targeting cancer epigenetics: linking basic biology to clinical medicine. Adv Drug Deliv Rev. 2015;95:56–64. 10.1016/j.addr.2015.10.006. 26494398 10.1016/j.addr.2015.10.006

[29] Villicana S, Bell JT. Genetic impacts on DNA methylation: research findings and future perspectives. Genome Biol. 2021;22(1):127. 10.1186/s13059-021-02347-6. 33931130 10.1186/s13059-021-02347-6 PMC8086086

[30] Dawson MA, Kouzarides T. Cancer epigenetics: from mechanism to therapy. Cell. 2012;150(1):12–27. 10.1016/j.cell.2012.06.013. 22770212 10.1016/j.cell.2012.06.013

[31] Alrehaili AA, et al. Evaluation of TET family gene expression and 5-hydroxymethylcytosine as potential epigenetic markers in non-small cell lung cancer. In Vivo. 2023;37(1):445–53. 10.21873/invivo.13098. 36593050 10.21873/invivo.13098 PMC9843776

[32] Casado-Pelaez M, Bueno-Costa A, Esteller M. Single cell cancer epigenetics. Trends Cancer. 2022;8(10):820–38. 10.1016/j.trecan.2022.06.005. 35821003 10.1016/j.trecan.2022.06.005

[33] Du P, et al. The miR-27a-3p/FTO axis modifies hypoxia-induced malignant behaviors of glioma cells. Acta Biochim Biophys Sin. 2023;55(1):103–16. 10.3724/abbs.2023002. 36718644 10.3724/abbs.2023002 PMC10157519

[34] Nishiyama A, Nakanishi M. Navigating the DNA methylation landscape of cancer. Trends Genet. 2021;37(11):1012–27. 10.1016/j.tig.2021.05.002. 34120771 10.1016/j.tig.2021.05.002

[35] Zhang L, Lu Q, Chang C. Epigenetics in health and disease. Adv Exp Med Biol. 2020;1253:3–55. 10.1007/978-981-15-3449-2_1. 32445090 10.1007/978-981-15-3449-2_1

[36] Jubber I, et al. Epidemiology of bladder cancer in 2023: a systematic review of risk factors. Eur Urol. 2023;84(2):176–90. 10.1016/j.eururo.2023.03.029. 37198015 10.1016/j.eururo.2023.03.029

[37] Moller L, et al. The epidemiology of colorectal cancer in younger and older patients. Dtsch Arztebl Int. 2023;120(16):277–83. 10.3238/arztebl.m2023.0041. 36919357 10.3238/arztebl.m2023.0041 PMC10304004

[38] Mederos N, et al. Gender-specific aspects of epidemiology, molecular genetics and outcome: lung cancer. ESMO Open. 2020;5(Suppl 4): e000796. 10.1136/esmoopen-2020-000796. 33148544 10.1136/esmoopen-2020-000796 PMC7643520

[39] Abood RA, Abdahmed KA, Mazyed SS. Epidemiology of different types of cancers reported in Basra Iraq. Sultan Qaboos Univ Med J. 2020;20(3):e295–300. 10.18295/squmj.2020.20.03.008. 33110644 10.18295/squmj.2020.20.03.008 PMC7574812

[40] Dogra P, et al. Translational modeling identifies synergy between nanoparticle-delivered miRNA-22 and standard-of-care drugs in triple-negative breast cancer. Pharm Res. 2022;39(3):511–28. 10.1007/s11095-022-03176-3. 35294699 10.1007/s11095-022-03176-3 PMC8986735

[41] Perepelyuk M, et al. Aptamer-hybrid nanoparticle bioconjugate efficiently delivers miRNA-29b to non-small-cell lung cancer cells and inhibits growth by downregulating essential oncoproteins. Int J Nanomed. 2016;11:3533–44. 10.2147/IJN.S110488. 10.2147/IJN.S110488 PMC4970448 27555773

[42] Qu A, et al. Quantitative zeptomolar imaging of miRNA cancer markers with nanoparticle assemblies. Proc Natl Acad Sci U S A. 2019;116(9):3391–400. 10.1073/pnas.1810764116. 30808736 10.1073/pnas.1810764116 PMC6397542

[43] Roberti A, et al. Epigenetics in cancer therapy and nanomedicine. Clin Epigenetics. 2019;11(1):81. 10.1186/s13148-019-0675-4. 31097014 10.1186/s13148-019-0675-4 PMC6524244

[44] Miranda Furtado CL, et al. Epidrugs: targeting epigenetic marks in cancer treatment. Epigenetics. 2019;14(12):1164–76. 10.1080/15592294.2019.1640546. 31282279 10.1080/15592294.2019.1640546 PMC6791710

[45] Nebbioso A, et al. Cancer epigenetics: moving forward. PLoS Genet. 2018;14(6): e1007362. 10.1371/journal.pgen.1007362. 29879107 10.1371/journal.pgen.1007362 PMC5991666

[46] Villanueva L, Alvarez-Errico D, Esteller M. The contribution of epigenetics to cancer immunotherapy. Trends Immunol. 2020;41(8):676–91. 10.1016/j.it.2020.06.002. 32622854 10.1016/j.it.2020.06.002

[47] Sun W, et al. Catalytic domain-dependent and -independent transcriptional activities of the tumour suppressor histone H3K27 demethylase UTX/KDM6A in specific cancer types. Epigenetics. 2023;18(1):2222245. 10.1080/15592294.2023.2222245. 37300822 10.1080/15592294.2023.2222245 PMC10259304

[48] Usui G, et al. DNA methylation and genetic aberrations in gastric cancer. Digestion. 2021;102(1):25–32. 10.1159/000511243. 33070127 10.1159/000511243

[49] Fabregat A, et al. Reactome diagram viewer: data structures and strategies to boost performance. Bioinformatics. 2018;34(7):1208–14. 10.1093/bioinformatics/btx752. 29186351 10.1093/bioinformatics/btx752 PMC6030826

[50] Chao YL, Pecot CV. Targeting epigenetics in lung cancer. Cold Spring Harb Perspect Med. 2021. 10.1101/cshperspect.a038000. 32900703 10.1101/cshperspect.a038000 PMC8168531

[51] Murata A, et al. TET family proteins and 5-hydroxymethylcytosine in esophageal squamous cell carcinoma. Oncotarget. 2015;6(27):23372–82. 10.18632/oncotarget.4281. 26093090 10.18632/oncotarget.4281 PMC4695124

[52] Zhang Y, et al. m6A modification in RNA: biogenesis, functions and roles in gliomas. J Exp Clin Cancer Res. 2020;39(1):192. 10.1186/s13046-020-01706-8. 32943100 10.1186/s13046-020-01706-8 PMC7500025

[53] Zhao Z, Shilatifard A. Epigenetic modifications of histones in cancer. Genome Biol. 2019;20(1):245. 10.1186/s13059-019-1870-5. 31747960 10.1186/s13059-019-1870-5 PMC6868810

[54] Liu Y, Zhu J, Ding L. Involvement of RNA methylation modification patterns mediated by m7G, m6A, m5C and m1A regulators in immune microenvironment regulation of Sjogren’s syndrome. Cell Signal. 2023;106:110650. 10.1016/j.cellsig.2023.110650. 36935085 10.1016/j.cellsig.2023.110650

[55] Veeck J, Esteller M. Breast cancer epigenetics: from DNA methylation to microRNAs. J Mammary Gland Biol Neoplasia. 2010;15(1):5–17. 10.1007/s10911-010-9165-1. 20101446 10.1007/s10911-010-9165-1 PMC2824126

[56] Shao D, et al. An m6A/m5C/m1A/m7G-Related Long non-coding RNA signature to predict prognosis and immune features of glioma. Front Genet. 2022;13:903117. 10.3389/fgene.2022.903117. 35692827 10.3389/fgene.2022.903117 PMC9178125

[57] Li D, et al. The m6A/m5C/m1A regulated gene signature predicts the prognosis and correlates with the immune status of hepatocellular carcinoma. Front Immunol. 2022;13:918140. 10.3389/fimmu.2022.918140. 35833147 10.3389/fimmu.2022.918140 PMC9272990

[58] Saliminejad K, et al. An overview of microRNAs: biology, functions, therapeutics, and analysis methods. J Cell Physiol. 2019;234(5):5451–65. 10.1002/jcp.27486. 30471116 10.1002/jcp.27486

[59] Lin S, Gregory RI. MicroRNA biogenesis pathways in cancer. Nat Rev Cancer. 2015;15(6):321–33. 10.1038/nrc3932. 25998712 10.1038/nrc3932 PMC4859809

[60] Yague-Sanz C, et al. Co-transcriptional RNA cleavage by Drosha homolog Pac1 triggers transcription termination in fission yeast. Nucleic Acids Res. 2021;49(15):8610–24. 10.1093/nar/gkab654. 34352089 10.1093/nar/gkab654 PMC8421224

[61] Abou Zeid LY, et al. Caspase-mediated cleavage of miRNA processing proteins Drosha, DGCR8, Dicer, and TRBP2 in heat-shocked cells and its inhibition by HSP70 overexpression. Cell Stress Chaperones. 2022;27(1):11–25. 10.1007/s12192-021-01242-0. 34719748 10.1007/s12192-021-01242-0 PMC8821752

[62] Calado A, et al. Exportin-5-mediated nuclear export of eukaryotic elongation factor 1A and tRNA. EMBO J. 2002;21(22):6216–24. 10.1093/emboj/cdf620. 12426393 10.1093/emboj/cdf620 PMC137209

[63] Lee YY, Kim H, Kim VN. Sequence determinant of small RNA production by DICER. Nature. 2023;615(7951):323–30. 10.1038/s41586-023-05722-4. 36813957 10.1038/s41586-023-05722-4

[64] Neumeier J, Meister G. siRNA specificity: RNAi mechanisms and strategies to reduce off-target effects. Front Plant Sci. 2020;11:526455. 10.3389/fpls.2020.526455. 33584737 10.3389/fpls.2020.526455 PMC7876455

[65] Nakanishi K. Anatomy of four human Argonaute proteins. Nucleic Acids Res. 2022;50(12):6618–38. 10.1093/nar/gkac519. 35736234 10.1093/nar/gkac519 PMC9262622

[66] Sun P, et al. MiR-34a inhibits cell proliferation and induces apoptosis in human nasopharyngeal carcinoma by targeting lncRNA MCM3AP-AS1. Cancer Manag Res. 2020;12:4799–806. 10.2147/CMAR.S245520. 32606969 10.2147/CMAR.S245520 PMC7319531

[67] Khani-Habibabadi F, et al. Hotair and Malat1 long noncoding RNAs regulate Bdnf expression and oligodendrocyte precursor cell differentiation. Mol Neurobiol. 2022;59(7):4209–22. 10.1007/s12035-022-02844-0. 35499794 10.1007/s12035-022-02844-0

[68] Sun H, et al. Ultrasensitive miRNA-21 biosensor based on Zn(TCPP) PET-RAFT polymerization signal amplification and multiple logic gate molecular recognition. ACS Appl Mater Interfaces. 2023;15(14):17716–25. 10.1021/acsami.3c02428. 36988387 10.1021/acsami.3c02428

[69] Gunawan RR, Astuti I, Danarto HR. miRNA-21 as high potential prostate cancer biomarker in prostate cancer patients in Indonesia. Asian Pac J Cancer Prev. 2023;24(3):1095–9. 10.31557/APJCP.2023.24.3.1095. 36974566 10.31557/APJCP.2023.24.3.1095 PMC10334082

[70] Zubrzycka A, et al. The expression of TGF-beta1, SMAD3, ILK and miRNA-21 in the ectopic and eutopic endometrium of women with endometriosis. Int J Mol Sci. 2023. 10.3390/ijms24032453. 36768775 10.3390/ijms24032453 PMC9917033

[71] Wu D, et al. In situ detection of miRNA-21 in MCF-7 cell-derived extracellular vesicles using the red blood cell membrane vesicle strategy. Chem Commun. 2023;59(14):1987–90. 10.1039/d2cc05954a. 10.1039/d2cc05954a 36723001

[72] Zhang Y, Mi Y, He C. 2-methoxyestradiol restrains non-small cell lung cancer tumorigenesis through regulating circ_0010235/miR-34a-5p/NFAT5 axis. Thorac Cancer. 2023;14(22):2105–15. 10.1111/1759-7714.14993. 37439026 10.1111/1759-7714.14993 PMC10396792

[73] Bayraktar R, Van Roosbroeck K. miR-155 in cancer drug resistance and as target for miRNA-based therapeutics. Cancer Metastasis Rev. 2018;37(1):33–44. 10.1007/s10555-017-9724-7. 29282605 10.1007/s10555-017-9724-7

[74] Watson KL, et al. The miR-200b/200a/429 cluster prevents metastasis and induces dormancy in a murine claudin-low mammary tumor cell line. Exp Cell Res. 2018;369(1):17–26. 10.1016/j.yexcr.2018.04.024. 29702103 10.1016/j.yexcr.2018.04.024

[75] Kuo G, Wu CY, Yang HY. MiR-17-92 cluster and immunity. J Formos Med Assoc. 2019;118(1 Pt 1):2–6. 10.1016/j.jfma.2018.04.013. 29857952 10.1016/j.jfma.2018.04.013

[76] Akamine PS, et al. Age-related increase of let-7 family microRNA in rat retina and vitreous. Exp Eye Res. 2021;204:108434. 10.1016/j.exer.2020.108434. 33412132 10.1016/j.exer.2020.108434

[77] Zhang R, et al. METTL3 mediates Ang-II-induced cardiac hypertrophy through accelerating pri-miR-221/222 maturation in an m6A-dependent manner. Cell Mol Biol Lett. 2022;27(1):55. 10.1186/s11658-022-00349-1. 35836108 10.1186/s11658-022-00349-1 PMC9284900

[78] Tian XP, et al. Acidic microenvironment up-regulates exosomal miR-21 and miR-10b in early-stage hepatocellular carcinoma to promote cancer cell proliferation and metastasis. Theranostics. 2019;9(7):1965–79. 10.7150/thno.30958. 31037150 10.7150/thno.30958 PMC6485281

[79] Harati-Sadegh M, et al. Relationship between miR-143/145 cluster variations and cancer risk: proof from a Meta-analysis. Nucleosides Nucleotides Nucl Acids. 2021;40(5):578–91. 10.1080/15257770.2021.1916030. 10.1080/15257770.2021.1916030 33980135

[80] Li ZY, et al. c-Myc-activated intronic miR-210 and lncRNA MIR210HG synergistically promote the metastasis of gastric cancer. Cancer Lett. 2022;526:322–34. 10.1016/j.canlet.2021.11.006. 34767926 10.1016/j.canlet.2021.11.006

[81] Soofiyani SR, et al. Prognostic value and biological role of miR-126 in breast cancer. Microrna. 2022;11(2):95–103. 10.2174/1876402914666220428123203. 35507794 10.2174/1876402914666220428123203

[82] Han J, et al. METTL3 promote tumor proliferation of bladder cancer by accelerating pri-miR221/222 maturation in m6A-dependent manner. Mol Cancer. 2019;18(1):110. 31228940 10.1186/s12943-019-1036-9 PMC6588935

[83] Bi J, et al. Circ-BPTF promotes bladder cancer progression and recurrence through the miR-31-5p/RAB27A axis. Aging. 2018;10(8):1964–76. 10.18632/aging.101520. 30103209 10.18632/aging.101520 PMC6128440

[84] Wang Y, et al. The role of miRNA-29 family in cancer. Eur J Cell Biol. 2013;92(3):123–8. 10.1016/j.ejcb.2012.11.004. 23357522 10.1016/j.ejcb.2012.11.004

[85] Costa-Pinheiro P, et al. Diagnostic and prognostic epigenetic biomarkers in cancer. Epigenomics. 2015;7(6):1003–15. 10.2217/epi.15.56. 26479312 10.2217/epi.15.56

[86] Grady WM, Yu M, Markowitz SD. Epigenetic alterations in the gastrointestinal tract: current and emerging use for biomarkers of cancer. Gastroenterology. 2021;160(3):690–709. 10.1053/j.gastro.2020.09.058. 33279516 10.1053/j.gastro.2020.09.058 PMC7878343

[87] Zafon C, et al. DNA methylation in thyroid cancer. Endocr Relat Cancer. 2019;26(7):R415–39. 10.1530/ERC-19-0093. 31035251 10.1530/ERC-19-0093

[88] Okugawa Y, Grady WM, Goel A. Epigenetic alterations in colorectal cancer: emerging biomarkers. Gastroenterology. 2015;149(5):1204-1225 e12. 10.1053/j.gastro.2015.07.011. 26216839 10.1053/j.gastro.2015.07.011 PMC4589488

[89] Salem ME, et al. Relationship between MLH1, PMS2, MSH2 and MSH6 gene-specific alterations and tumor mutational burden in 1057 microsatellite instability-high solid tumors. Int J Cancer. 2020;147(10):2948–56. 10.1002/ijc.33115. 32449172 10.1002/ijc.33115 PMC7530095

[90] Butler M, et al. MGMT status as a clinical biomarker in glioblastoma. Trends Cancer. 2020;6(5):380–91. 10.1016/j.trecan.2020.02.010. 32348734 10.1016/j.trecan.2020.02.010 PMC7315323

[91] Whitmore MA, et al. Epigenetic regulation of host defense peptide synthesis: synergy between histone deacetylase inhibitors and DNA/Histone methyltransferase inhibitors. Front Immunol. 2022;13:874706. 10.3389/fimmu.2022.874706. 35529861 10.3389/fimmu.2022.874706 PMC9074817

[92] Hu C, et al. DNA methyltransferase inhibitors combination therapy for the treatment of solid tumor: mechanism and clinical application. Clin Epigenetics. 2021;13(1):166. 10.1186/s13148-021-01154-x. 34452630 10.1186/s13148-021-01154-x PMC8394595

[93] Sarhadi VK, Armengol G. Molecular biomarkers in cancer. Biomolecules. 2022. 10.3390/biom12081021. 35892331 10.3390/biom12081021 PMC9331210

[94] Sapienza C, Issa JP. Diet, nutrition, and cancer epigenetics. Annu Rev Nutr. 2016;36:665–81. 10.1146/annurev-nutr-121415-112634. 27022771 10.1146/annurev-nutr-121415-112634

[95] Acar Y, Akbulut G. Nutritional epigenetics and phytochemicals in cancer formation. J Am Nutr Assoc. 2023;42(7):700–5. 10.1080/27697061.2022.2147106. 36416668 10.1080/27697061.2022.2147106

[96] Sharma S, Kelly TK, Jones PA. Epigenetics in cancer. Carcinogenesis. 2010;31(1):27–36. 10.1093/carcin/bgp220. 19752007 10.1093/carcin/bgp220 PMC2802667

[97] Sigalotti L, et al. Epigenetic drugs as immunomodulators for combination therapies in solid tumors. Pharmacol Ther. 2014;142(3):339–50. 10.1016/j.pharmthera.2013.12.015. 24384533 10.1016/j.pharmthera.2013.12.015

[98] Flavahan WA, Gaskell E, Bernstein BE. Epigenetic plasticity and the hallmarks of cancer. Science. 2017. 10.1126/science.aal2380. 28729483 10.1126/science.aal2380 PMC5940341

[99] Feng S, De Carvalho DD. Clinical advances in targeting epigenetics for cancer therapy. FEBS J. 2022;289(5):1214–39. 10.1111/febs.15750. 33545740 10.1111/febs.15750

[100] Liu Z, et al. A new trend in cancer treatment: the combination of epigenetics and immunotherapy. Front Immunol. 2022;13:809761. 10.3389/fimmu.2022.809761. 35140720 10.3389/fimmu.2022.809761 PMC8818678

[101] Oieni J, et al. Nano-ghosts: novel biomimetic nano-vesicles for the delivery of antisense oligonucleotides. J Control Release. 2021;333:28–40. 10.1016/j.jconrel.2021.03.018. 33741386 10.1016/j.jconrel.2021.03.018

[102] Wagner E. Tumor-targeted delivery of Anti-microRNA for cancer therapy: pHLIP is key. Angew Chem Int Ed Engl. 2015;54(20):5824–6. 10.1002/anie.201502146. 25892205 10.1002/anie.201502146

[103] Bernardo BC, et al. miRNA therapeutics: a new class of drugs with potential therapeutic applications in the heart. Future Med Chem. 2015;7(13):1771–92. 10.4155/fmc.15.107. 26399457 10.4155/fmc.15.107

[104] Cheng CJ, et al. MicroRNA silencing for cancer therapy targeted to the tumour microenvironment. Nature. 2015;518(7537):107–10. 10.1038/nature13905. 25409146 10.1038/nature13905 PMC4367962

[105] Clausse V, et al. Thyclotides, tetrahydrofuran-modified peptide nucleic acids that efficiently penetrate cells and inhibit microRNA-21. Nucleic Acids Res. 2022;50(19):10839–56. 10.1093/nar/gkac864. 36215040 10.1093/nar/gkac864 PMC9638920

[106] Maryam M, Naemi M, Hasani SS. A comprehensive review on oncogenic miRNAs in breast cancer. J Genet. 2021. 10.1007/s12041-021-01265-7. 33764337

[107] Lennox KA, Vakulskas CA, Behlke MA. Non-nucleotide modification of anti-miRNA oligonucleotides. Methods Mol Biol. 2017;1517:51–69. 10.1007/978-1-4939-6563-2_3. 27924473 10.1007/978-1-4939-6563-2_3

[108] El-Mancy SS, et al. Enhancement of bottle gourd oil activity via optimized self-dispersing lipid formulations (SDLFs) to mitigate isoproterenol-evoked cardiac toxicity in rats via modulating BMP, MMP2, and miRNA-21 and miRNA-23a genes’ expression. Molecules. 2023. 10.3390/molecules28166168. 37630419 10.3390/molecules28166168 PMC10458851

[109] Chen M, et al. CRISPR-Cas9 for cancer therapy: opportunities and challenges. Cancer Lett. 2019;447:48–55. 10.1016/j.canlet.2019.01.017. 30684591 10.1016/j.canlet.2019.01.017

[110] Lin S, et al. An in vivo CRISPR screening platform for prioritizing therapeutic targets in AML. Cancer Discov. 2022;12(2):432–49. 10.1158/2159-8290.CD-20-1851. 34531254 10.1158/2159-8290.CD-20-1851 PMC8831447

[111] Kurata M, et al. CRISPR/Cas9 library screening for drug target discovery. J Hum Genet. 2018;63(2):179–86. 10.1038/s10038-017-0376-9. 29158600 10.1038/s10038-017-0376-9

[112] Stadtmauer EA, et al. CRISPR-engineered T cells in patients with refractory cancer. Science. 2020. 10.1038/s10038-017-0376-9. 32029687 10.1126/science.aba7365 PMC11249135

[113] Huang S, et al. Genome-wide CRISPR/Cas9 library screening identified that DUSP4 deficiency induces lenvatinib resistance in hepatocellular carcinoma. Int J Biol Sci. 2022;18(11):4357–71. 10.7150/ijbs.69969. 35864956 10.7150/ijbs.69969 PMC9295068

[114] Jones PA, Takai D. The role of DNA methylation in mammalian epigenetics. Science. 2001;293(5532):1068–70. 10.1126/science.1063852. 11498573 10.1126/science.1063852

[115] Buocikova V, et al. Epigenetics in breast cancer therapy-new strategies and future nanomedicine perspectives. Cancers. 2020. 10.3390/cancers12123622. 33287297 10.3390/cancers12123622 PMC7761669

[116] Ahmadzada T, Reid G, McKenzie DR. Fundamentals of siRNA and miRNA therapeutics and a review of targeted nanoparticle delivery systems in breast cancer. Biophys Rev. 2018;10(1):69–86. 10.1007/s12551-017-0392-1. 29327101 10.1007/s12551-017-0392-1 PMC5803180

[117] Sun Z, et al. In vivo multimodality imaging of miRNA-16 iron nanoparticle reversing drug resistance to chemotherapy in a mouse gastric cancer model. Nanoscale. 2014;6(23):14343–53. 10.1039/c4nr03003f. 25327162 10.1039/c4nr03003f

[118] Sendi H, et al. Nanoparticle delivery of miR-122 inhibits colorectal cancer liver metastasis. Cancer Res. 2022;82(1):105–13. 10.1158/0008-5472.CAN-21-2269. 34753773 10.1158/0008-5472.CAN-21-2269 PMC8732321

[119] Sartor O, et al. Lutetium-177-PSMA-617 for metastatic castration-resistant prostate cancer. N Engl J Med. 2021;385(12):1091–103. 10.1056/NEJMoa2107322. 34161051 10.1056/NEJMoa2107322 PMC8446332

[120] Lee BS, et al. Improving theranostic gallium-68/lutetium-177-labeled PSMA inhibitors with an albumin binder for prostate cancer. Mol Cancer Ther. 2021;20(12):2410–9. 10.1158/1535-7163.MCT-21-0251. 34725194 10.1158/1535-7163.MCT-21-0251

[121] Rieger K, et al. 90-yttrium-ibritumomab tiuxetan as first-line treatment for follicular lymphoma: updated efficacy and safety results at an extended median follow-up of 9.6 years. Ann Hematol. 2022;101(4):781–8. 10.1007/s00277-022-04781-3. 35150296 10.1007/s00277-022-04781-3 PMC8913448

[122] Jimenez C, Nunez R, Wendt R. High-specific-activity iodine 131 metaiodobenzylguanidine for the treatment of metastatic pheochromocytoma or paraganglioma: a novel therapy for an orphan disease. Curr Opin Endocrinol Diabetes Obes. 2020;27(3):162–9. 10.1097/MED.0000000000000544. 32250976 10.1097/MED.0000000000000544

[123] Hyvakka A, et al. Radium-223 dichloride treatment in metastatic castration-resistant prostate cancer in Finland: a real-world evidence multicenter study. Cancer Med. 2023;12(4):4064–76. 10.1002/cam4.5262. 36156455 10.1002/cam4.5262 PMC9972699

[124] Havlena GT, et al. Cure of micrometastatic B-cell lymphoma in a SCID mouse model using (213)Bi-Anti-CD20 monoclonal antibody. J Nucl Med. 2023;64(1):109–16. 10.2967/jnumed.122.263962. 35981897 10.2967/jnumed.122.263962 PMC9841256

[125] Yoneshima Y, et al. Phase 3 trial comparing nanoparticle albumin-bound paclitaxel with docetaxel for previously treated advanced NSCLC. J Thorac Oncol. 2021;16(9):1523–32. 10.1016/j.jtho.2021.03.027. 33915251 10.1016/j.jtho.2021.03.027

[126] Song H, et al. 64Cu-DOTATATE uptake in a pulmonary hamartoma. Clin Nucl Med. 2023;48(1):58–60. 10.1097/RLU.0000000000004390. 36469060 10.1097/RLU.0000000000004390

[127] Jungels C, Karfis I. 131I-metaiodobenzylguanidine and peptide receptor radionuclide therapy in pheochromocytoma and paraganglioma. Curr Opin Oncol. 2021;33(1):33–9. 10.1097/CCO.0000000000000691. 33093336 10.1097/CCO.0000000000000691

[128] Goins B, Bao A, Phillips WT. Techniques for loading technetium-99m and rhenium-186/188 radionuclides into preformed liposomes for diagnostic imaging and radionuclide therapy. Methods Mol Biol. 2017;1522:155–78. 10.1007/978-1-4939-6591-5_13. 27837538 10.1007/978-1-4939-6591-5_13

[129] Strosberg JR, et al. (177)Lu-Dotatate plus long-acting octreotide versus high-dose long-acting octreotide in patients with midgut neuroendocrine tumours (NETTER-1): final overall survival and long-term safety results from an open-label, randomised, controlled, phase 3 trial. Lancet Oncol. 2021;22(12):1752–63. 10.1016/S1470-2045(21)00572-6. 34793718 10.1016/S1470-2045(21)00572-6

[130] Reda M, et al. Development of a nanoparticle-based immunotherapy targeting PD-L1 and PLK1 for lung cancer treatment. Nat Commun. 2022;13(1):4261. 10.1038/s41467-022-31926-9. 35871223 10.1038/s41467-022-31926-9 PMC9308817

[131] Li B, et al. Nanoparticle-based combination therapy enhances fulvestrant efficacy and overcomes tumor resistance in ER-positive breast cancer. Cancer Res. 2023;83(17):2924–37. 10.1158/0008-5472.CAN-22-3559. 37326467 10.1158/0008-5472.CAN-22-3559

[132] Elzayat EM, et al. Enhanced codelivery of gefitinib and azacitidine for treatment of metastatic-resistant lung cancer using biodegradable lipid nanoparticles. Materials. 2023. 10.3390/ma16155364. 37570067 10.3390/ma16155364 PMC10419431

[133] Mehrotra N, et al. Polylactic acid based polymeric nanoparticle mediated co-delivery of navitoclax and decitabine for cancer therapy. Nanomedicine. 2023;47:102627. 10.1016/j.nano.2022.102627. 36410699 10.1016/j.nano.2022.102627

[134] Zhu S, et al. A nanoparticle depot formulation of 4-(N)-stearoyl gemcitabine shows a strong anti-tumour activity. J Pharm Pharmacol. 2013;65(2):236–42. 10.1111/j.2042-7158.2012.01599.x. 23278691 10.1111/j.2042-7158.2012.01599.x PMC3539214

[135] Jueliger S, et al. Efficacy and epigenetic interactions of novel DNA hypomethylating agent guadecitabine (SGI-110) in preclinical models of hepatocellular carcinoma. Epigenetics. 2016;11(10):709–20. 10.1080/15592294.2016.1214781. 27646854 10.1080/15592294.2016.1214781 PMC5094635

[136] Marczak M, et al. A new face of the old gene: deletion of the PssA, encoding monotopic inner membrane phosphoglycosyl transferase in rhizobium leguminosarum, leads to diverse phenotypes that could be attributable to downstream effects of the lack of exopolysaccharide. Int J Mol Sci. 2023. 10.3390/ijms24021035. 36674551 10.3390/ijms24021035 PMC9860679

[137] Ruan H, et al. A dual-bioresponsive drug-delivery depot for combination of epigenetic modulation and immune checkpoint blockade. Adv Mater. 2019;31(17): e1806957. 10.1002/adma.201806957. 30856290 10.1002/adma.201806957

[138] Meka AK, et al. Enhanced solubility, permeability and anticancer activity of vorinostat using tailored mesoporous silica nanoparticles. Pharmaceutics. 2018. 10.3390/pharmaceutics10040283. 30562958 10.3390/pharmaceutics10040283 PMC6321298

[139] Kumar P, et al. Co-delivery of vorinostat and etoposide via disulfide cross-linked biodegradable polymeric nanogels: synthesis, characterization, biodegradation, and anticancer activity. AAPS PharmSciTech. 2018;19(2):634–47. 10.1208/s12249-017-0863-5. 28948528 10.1208/s12249-017-0863-5

[140] Shan S, et al. Functionalized macrophage exosomes with panobinostat and PPM1D-siRNA for diffuse intrinsic pontine gliomas therapy. Adv Sci. 2022;9(21): e2200353. 10.1002/advs.202200353. 10.1002/advs.202200353 PMC9313473 35585670

[141] Nunn AD, et al. The histone deacetylase inhibiting drug Entinostat induces lipid accumulation in differentiated HepaRG cells. Sci Rep. 2016;6:28025. 10.1038/srep28025. 27320682 10.1038/srep28025 PMC4913258

[142] Hartwig O, et al. Leaky gut model of the human intestinal mucosa for testing siRNA-based nanomedicine targeting JAK1. J Control Release. 2022;345:646–60. 10.1016/j.jconrel.2022.03.037. 35339579 10.1016/j.jconrel.2022.03.037 PMC9168449

[143] Subhan MA, Filipczak N, Torchilin VP. Advances with lipid-based nanosystems for siRNA delivery to breast cancers. Pharmaceuticals. 2023. 10.3390/ph16070970. 37513882 10.3390/ph16070970 PMC10386415

[144] Scott LJ. Givosiran: first approval. Drugs. 2020;80(3):335–9. 10.1007/s40265-020-01269-0. 32034693 10.1007/s40265-020-01269-0

[145] Viiavabaskar MS, Obier N, et al. Integrated analyses of chromatin accessibility and gene expression data for elucidating the transcriptional regulatory mechanisms during early hematopoietic development in mouse. Epigenetics Chromatin. 2013;6:1–2. 10.1186/1756-8935-6-S1-P50.23289424

[146] Disatham J, et al. Changes in DNA methylation hallmark alterations in chromatin accessibility and gene expression for eye lens differentiation. Epigenetics Chromatin. 2022;15(1):8. 10.1186/s13072-022-00440-z. 35246225 10.1186/s13072-022-00440-z PMC8897925

[147] Decombe S, et al. Epigenetic rewriting at centromeric DNA repeats leads to increased chromatin accessibility and chromosomal instability. Epigenetics Chromatin. 2021;14:1–7. 10.1186/s13072-021-00410-x. 34321103 10.1186/s13072-021-00410-x PMC8317386

[148] Wang M, Sunkel BD, Ray WC, Stanton BZ, Wang M, Sunkel BD, Ray WC, Stanton BZ. Chromatin structure in cancer. BMC Mol Cell Biol. 2022;23(1):35. 10.1186/s12860-022-00433-6. 35902807 10.1186/s12860-022-00433-6 PMC9331575

[149] Siggens L, Cordeddu L, Rönnerblad M, et al. Transcription-coupled recruitment of human CHD1 and CHD2 influences chromatin accessibility and histone H3 and H3. 3 occupancy at active chromatin regions. Epigenetics Chromatin. 2015;8:1–4. 10.1186/1756-8935-8-4. 25621013 10.1186/1756-8935-8-4 PMC4305392

[150] Jiang JJ, Cheng LH, Wu H, et al. Insights into long noncoding RNAs of naked mole rat (Heterocephalus glaber) and their potential association with cancer resistance. Epigenetics Chromatin. 2016;9:51. 10.1186/s13072-016-0101-5. 27833660 10.1186/s13072-016-0101-5 PMC5103457

[151] Mahadevan IA, Kumar S, Rao MRS. Linker histone variant H1t is closely associated with repressed repeat-element chromatin domains in pachytene spermatocytes. Epigenetics Chromatin. 2020;13:9. 10.1186/s13072-020-00335-x. 32131873 10.1186/s13072-020-00335-x PMC7057672

[152] Jemal A, Center MM, DeSantis C, Ward EM. Global patterns of cancer incidence and mortality rates and trends. Cancer Epidemiol Biomark Prev. 2010;19(8):1893–907. 10.1158/1055-9965.EPI-10-0437 20647400

[153] Leiter A, Veluswamy RR, Wisnivesky JP. The global burden of lung cancer: current status and future trends. Nat Rev Clin Oncol. 2023;20(9):624–39. 37479810 10.1038/s41571-023-00798-3

[154] Torre LA, Siegel RL, Jemal A. Lung cancer statistics. Lung Cancer Personal Med Curr Knowl Ther. 2016;893:1–9. 10.1007/978-3-319-24223-1_1 26667336

[155] Lei S, Zheng R, Zhang S, et al. Global patterns of breast cancer incidence and mortality: a population-based cancer registry data analysis from 2000 to 2020. Cancer Commun. 2021;41(11):1183–94. 10.1002/cac2.12207 PMC8626596 34399040

[156] Sharma R. A comparative examination of colorectal cancer burden in European Union, 1990–2019: estimates from global burden of disease 2019 Study. Int J Clin Oncol. 2022;27:1309–20. 10.1007/s10147-022-02182-0. 35590123 10.1007/s10147-022-02182-0

[157] Lu B, Li N, Luo C, Y., et al. Colorectal cancer incidence and mortality: the current status, temporal trends and their attributable risk factors in 60 countries in 2000–2019. Chin Med J. 2021;134(16):1941–51. 10.1097/CM9.0000000000001619. 34238851 10.1097/CM9.0000000000001619 PMC8382382

[158] Ilic M, Ilic I. Epidemiology of stomach cancer. World J Gastroenterol. 2022;28(12):1187. 10.3748/wjg.v28.i12.1187. 35431510 10.3748/wjg.v28.i12.1187 PMC8968487

[159] Rumgay H, Arnold M, Ferlay J, et al. Global burden of primary liver cancer in 2020 and predictions to 2040. J Hepatol. 2022;77(6):1598–606. 10.1016/j.jhep.2022.08.021. 36208844 10.1016/j.jhep.2022.08.021 PMC9670241

[160] Subhan MA, Choudhury KP, Neogi N. Advances with molecular nanomaterials in industrial manufacturing applications. Nanomanufacturing. 2021;1(2):75–97. 10.3390/nanomanufacturing1020008.

[161] Fadeel B, Feliu N, Vogt C, Abdelmonem AM, Parak WJ. Bridge over troubled waters: understanding the synthetic and biological identities of engineered nanomaterials. Wiley Interdiscip Rev Nanomed Nanobiotechnol. 2013;5(2):111–29. 10.1002/wnan.1206. 23335558 10.1002/wnan.1206

[162] Cheng X, Xie Q, Sun Y. Advances in nanomaterial-based targeted drug delivery systems. Front Bioeng Biotechnol. 2023;11:1177151. 10.3389/fbioe.2023.1177151. 37122851 10.3389/fbioe.2023.1177151 PMC10133513

[163] Chehelgerdi M, Chehelgerdi M, Allela OQB, et al. Progressing nanotechnology to improve targeted cancer treatment: overcoming hurdles in its clinical implementation. Mol Cancer. 2023;22(1):169. 10.1186/s12943-023-01865-0. 37814270 10.1186/s12943-023-01865-0 PMC10561438

[164] Ghosh S, Ghosh S. Nanomedicine for overcoming drug resistance in cancer. J Control Release. 2017;266:169–90. 10.1016/j.jconrel.2017.09.007 28893609

[165] Yang Y, Zhang J, Xia F, Zhang Z, Qin S. Recent advances in stimuli-responsive drug delivery systems for cancer therapy. J Drug Target. 2015;23(7–8):580–90.26453156

[166] Paliwal R, Babu RJ, Palakurthi S. Nanomedicine scale-up technologies: feasibilities and challenges. AAPS PharmSciTech. 2014;15(6):1527–34. 10.1208/s12249-014-0177-9. 25047256 10.1208/s12249-014-0177-9 PMC4245446

[167] Herdiana Y, Wathoni N, Shamsuddin S, Muchtaridi M. Scale-up polymeric-based nanoparticles drug delivery systems: development and challenges. OpenNano. 2022;7:100048. 10.1016/j.onano.2022.100048.

[168] Allan J, Belz S, Hoeveler A, et al. Regulatory landscape of nanotechnology and nanoplastics from a global perspective. Regul Toxicol Pharmacol. 2021;122:104885. 10.1016/j.yrtph.2021.104885. 33617940 10.1016/j.yrtph.2021.104885 PMC8121750

[169] Sun L, Liu H, Ye Y, et al. Smart nanoparticles for cancer therapy. Sig Transduct Target Ther. 2023;8:418. 10.1038/s41392-023-01642-x. 10.1038/s41392-023-01642-x PMC10622502 37919282

[170] Chudasama M, Goyary J. Nanostructured materials in food science: current progress and future prospects. Next Mater. 2024. 10.1016/j.nxmate.2024.100206.

[171] Pateiro M, Gómez B, Munekata PES, et al. Nanoencapsulation of promising bioactive compounds to improve their absorption, stability, functionality, and the appearance of the final food products. Molecules. 2021;26(6):1547. 10.3390/molecules26061547. 33799855 10.3390/molecules26061547 PMC7999092

[172] Elumalai K, Srinivasan S, Shanmugam A. Review of the efficacy of nanoparticle-based drug delivery systems for cancer treatment. Biomed Technol. 2024;5:109–22. 10.1016/j.bmt.2023.09.001.

[173] Egbuna C, Parmar VK, Jeevanandam J, et al. Toxicity of nanoparticles in biomedical application: nanotoxicology. J Toxicol. 2021;2021:9954443. 10.1155/2021/9954443. 34422042 10.1155/2021/9954443 PMC8376461

[174] Jaldurgam FF, Ahmad Z, Touati F. Synthesis and performance of large-scale cost-effective environment-friendly nanostructured thermoelectric materials. Nanomaterials. 2021;11(5):1091. 10.3390/nano11051091. 33922455 10.3390/nano11051091 PMC8146525

[175] Wasti S, Lee IH, Kim S, Lee JH, Kim H. Ethical and legal challenges in nanomedical innovations: a scoping review. Front Genet. 2023;14:1163392. 10.3389/fgene.2023.1163392. 37252668 10.3389/fgene.2023.1163392 PMC10213273

[176] Anselmo AC, Mitragotri S. Nanoparticles in the clinic. Bioeng Transl Med. 2016;1(1):10–29. 10.1002/btm2.10003. 29313004 10.1002/btm2.10003 PMC5689513

[177] Nguyen NHA, Falagan-Lotsch P. Mechanistic insights into the biological effects of engineered nanomaterials: a focus on gold nanoparticles. Int J Mol Sci. 2023;24(4):4109. 10.3390/ijms24044109. 36835521 10.3390/ijms24044109 PMC9963226

[178] Brzóska K, Sochanowicz B, Szczygieł M, et al. Silver nanoparticles induced changes in DNA methylation and histone H3 methylation in a mouse model of breast cancer. Materials. 2023;16(11):4163. 10.3390/ma16114163. 37297299 10.3390/ma16114163 PMC10254218

[179] Farzin A, Etesami SA, Quint J, Memic A, Tamayol A. Magnetic nanoparticles in cancer therapy and diagnosis. Adv Healthc Mater. 2020;9(9): e1901058. 10.1002/adhm.201901058. 32196144 10.1002/adhm.201901058 PMC7482193

[180] Zhang Y, Wang TH. Quantum dot enabled molecular sensing and diagnostics. Theranostics. 2012;2(7):631. 10.7150/thno.4308. 22916072 10.7150/thno.4308 PMC3425091

[181] Zare H, Ahmadi S, Ghasemi A, et al. Carbon nanotubes: smart drug/gene delivery carriers. Int J Nanomed. 2021;16:1681–706. 10.2147/IJN.S299448. 10.2147/IJN.S299448 PMC7936533 33688185

[182] Nsairat H, Khater D, Sayed U, Odeh F, Al Bawab A, Alshaer W. Liposomes: Structure, composition, types, and clinical applications. Heliyon. 2022;8(5): e09394. 10.1016/j.heliyon.2022.e09394. 35600452 10.1016/j.heliyon.2022.e09394 PMC9118483

[183] Gagliardi A, Giuliano E, Venkateswararao E, et al. Biodegradable polymeric nanoparticles for drug delivery to solid tumors. Front Pharmacol. 2021;12:601626. 10.3389/fphar.2021.601626. 33613290 10.3389/fphar.2021.601626 PMC7887387

[184] Zenze M, Daniels A, Singh M. Dendrimers as modifiers of inorganic nanoparticles for therapeutic delivery in cancer. Pharmaceutics. 2023;15(2):398. 10.3390/pharmaceutics15020398. 36839720 10.3390/pharmaceutics15020398 PMC9961584

[185] Živojević K, Mladenović M, Djisalov M, Mundzic M, Ruiz-Hernandez E, Gadjanski I, Knežević NŽ. Advanced mesoporous silica nanocarriers in cancer theranostics and gene editing applications. J Control Release. 2021;337:193–211. 10.1016/j.jconrel.2021.07.029. 34293320 10.1016/j.jconrel.2021.07.029

